# Forecasting severe respiratory disease hospitalizations using machine learning algorithms

**DOI:** 10.1186/s12911-024-02702-0

**Published:** 2024-10-09

**Authors:** Steffen Albrecht, David Broderick, Katharina Dost, Isabella Cheung, Nhung Nghiem, Milton Wu, Johnny Zhu, Nooriyan Poonawala-Lohani, Sarah Jamison, Damayanthi Rasanathan, Sue Huang, Adrian Trenholme, Alicia Stanley, Shirley Lawrence, Samantha Marsh, Lorraine Castelino, Janine Paynter, Nikki Turner, Peter McIntyre, Patricia Riddle, Cameron Grant, Gillian Dobbie, Jörg Simon Wicker

**Affiliations:** 1 University of Auckland, 20 Symonds Street, Auckland, 1010 New Zealand; 2 Australian National University, 131 Garran Rd, Acton, Canberra ACT 2601 Australia; 3 Institute of Environmental Science and Research, 34 Kenepuru Drive, Kenepuru, Porirua, 5022 New Zealand; 4 Health New Zealand Counties Manukau, Middlemore Hospital, 100 Hospital Road, Auckland, 2025 New Zealand; 5 Health New Zealand Te Toka Tumai Auckland, Auckland City Hospital, 2 Park Road, Auckland, 1023 New Zealand; 6 University of Otago, 362 Leith Street, Dunedin, 9016 New Zealand

**Keywords:** Forecasting healthcare burden, Seasonal epidemic, Influenza-like illness, Severe respiratory diseases, Forecasting, Flu prediction, Artificial intelligence, Machine learning, Probabilistic forecast

## Abstract

**Background:**

Forecasting models predicting trends in hospitalization rates have the potential to inform hospital management during seasonal epidemics of respiratory diseases and the associated surges caused by acute hospital admissions. Hospital bed requirements for elective surgery could be better planned if it were possible to foresee upcoming peaks in severe respiratory illness admissions. Forecasting models can also guide the use of intervention strategies to decrease the spread of respiratory pathogens and thus prevent local health system overload. In this study, we explore the capability of forecasting models to predict the number of hospital admissions in Auckland, New Zealand, within a three-week time horizon. Furthermore, we evaluate probabilistic forecasts and the impact on model performance when integrating laboratory data describing the circulation of respiratory viruses.

**Methods:**

The dataset used for this exploration results from active hospital surveillance, in which the World Health Organization Severe Acute Respiratory Infection (SARI) case definition was consistently used. This research nurse-led surveillance has been implemented in two public hospitals in Auckland and provides a systematic laboratory testing of SARI patients for nine respiratory viruses, including influenza, respiratory syncytial virus, and rhinovirus. The forecasting strategies used comprise automatic machine learning, one of the most recent generative pre-trained transformers, and established artificial neural network algorithms capable of univariate and multivariate forecasting.

**Results:**

We found that machine learning models compute more accurate forecasts in comparison to naïve seasonal models. Furthermore, we analyzed the impact of reducing the temporal resolution of forecasts, which decreased the model error of point forecasts and made probabilistic forecasting more reliable. An additional analysis that used the laboratory data revealed strong season-to-season variations in the incidence of respiratory viruses and how this correlates with total hospitalization cases. These variations could explain why it was not possible to improve forecasts by integrating this data.

**Conclusions:**

Active SARI surveillance and consistent data collection over time enable these data to be used to predict hospital bed utilization. These findings show the potential of machine learning as support for informing systems for proactive hospital management.

**Supplementary Information:**

The online version contains supplementary material available at 10.1186/s12911-024-02702-0.

## Introduction and background

Seasonal epidemics of respiratory infections challenge health systems worldwide [1–5]. Surges in acute hospital admissions caused by seasonal respiratory viral epidemics can require elective hospital admissions to be canceled at short notice. Consequently, this results in stress for the healthcare system, patients, and their families. To prevent these adverse outcomes, forecasting models can predict upcoming peaks in acute respiratory infection-related admissions and enable clinical leaders in hospitals to proactively manage medical resource utilization, staffing, and the scheduling of elective procedures. Moreover, forecasting allows policymakers to be better informed about applying interventions to reduce respiratory pathogen transmission rates and prevent local health system overload.

The application of machine learning to time series forecasting has increased in recent years due to its strong ability to achieve higher predictive accuracy in comparison to naïve models solely based on seasonal patterns and linear statistical methods such as ARIMA [6]. For this reason, several software frameworks offer algorithms for a range of time series analyses, including clustering, classification, and forecasting, applying concepts from tree-based and artificial neural network learning [7–10]. Other frameworks apply automated machine learning (AutoML) that, given a time series as input, systematically applies a collection of statistical methods and machine learning algorithms to explore which approach provides the best results on the data provided [11]. The user receives an automatically determined selection of the best algorithm or an ensemble output weighted by the performance of the algorithms used to predict future values for the time series provided. Such solutions are promising for the field of forecasting as they rely on the collective potential and complementary advantages of different algorithmic concepts.

Forecasting algorithms can be differentiated into two groups: *univariate* algorithms, using only one time series at a time and making use of statistical patterns in this time series to model future values, and *multivariate* algorithms that can integrate multiple time series to leverage statistical patterns across different time series integrated into a model that provides predictions for either one target time series (multivariate-to-univariate forecasting) or all series provided (multivariate-to-multivariate forecasting).

Multivariate forecasting is relevant as the predictive performance can be improved by providing additional information to the algorithm besides the target time series the model is trained for. For instance, weather information, such as temperature, precipitation, and humidity, has been integrated into several COVID-19 forecasting models developed for data from different countries [12–15]. Regarding influenza or influenza-like illness forecasting, there are approaches using weather data besides additional geographical information or leveraging internet search information, such as Google Trends [16–22]. These studies demonstrate that data integration coupled with machine learning can be highly beneficial as dedicated algorithms are able to extract complex patterns across different data sources to provide accurate forecasting models [23].

In this study, we investigate the potential of both univariate and multivariate forecasting algorithms for forecasting hospital admissions caused by respiratory infections with a severe disease progression monitored during the winter season in two hospitals in Auckland, New Zealand. The dataset we used for this study has been derived from the SHIVERS surveillance of hospitalizations, with this active surveillance system having consistently used the World Health Organization severe acute respiratory infection (SARI) case definition since the surveillance platform was established in 2012 [24]. This SARI surveillance data accurately describes the healthcare burden during seasonal epidemics of respiratory diseases as the SARI definition identifies patients with a specific combination of symptoms (cough and fever), recent onset of these symptoms (within the past ten days), and that require inpatient hospitalization [25].

The two participating hospitals in Auckland, New Zealand, provide secondary and tertiary healthcare to a population of more than 900,000 people, of which the socioeconomic and ethnic composition are broadly like the population of the whole country [25]. Moreover, the dataset provides a consistent laboratory component describing the result of real-time PCR protocols applied to test consenting SARI patients if they have been infected with one of nine respiratory viruses. These data describe which viruses circulate at daily resolution and can be integrated by a multivariate forecasting model. As the laboratory component encodes highly relevant information about the infectious dynamics of different viruses during the seasonal epidemics of respiratory diseases, we investigated whether it could aid the prediction of SARI hospitalization. In addition to the laboratory component, we expand the multivariate forecasting by using the SARI cases split into age groups representing infectious dynamics that differ between adults and children in different age groups.

We investigate a time window of 21 days for which the models are trained to provide a forecast at daily resolution. This time window is called the *forecasting horizon*. The forecasts were evaluated for each day on the horizon at daily resolution and at weekly resolution, using the average over seven days in the forecast. Furthermore, we include the evaluation of probabilistic forecasting, also called quantile forecasts, in our validation to assess how precise those quantiles, estimated by the forecasting algorithm, are. This is relevant as probabilistic forecasting can be used to create, in addition to the point forecast, a confidence interval describing the probability of observing the true value within this interval. Computing and visualizing model confidence is of crucial importance for decision-makers as it allows them to assess how much they should trust the model’s prediction.

A possible application scenario is to use forecasts within an information system in hospital management for the upcoming years, expecting that seasonal epidemics of respiratory diseases will have similar patterns to those observed prior to the COVID-19 pandemic. Therefore, we focus on data from eight years of this surveillance from 2012 to 2019, offering a perspective for using forecasting models in hospital management in winter seasons without strict social distancing rules as imposed during the pandemic era. Furthermore, this study serves as the groundwork for future studies that will be more concerned with the challenges that arise with data showing different patterns as it was collected during the COVID-19 pandemic in which social distancing rules and strict border closure policies were imposed by the New Zealand government.

This is the first study that applies machine learning-based forecasting to this SARI surveillance data. Besides other studies related to the forecasting of respiratory illness [12–23, 26], this study contributes to this research area by benchmarking a large selection of algorithms on a dataset with low daily incidence numbers, describing only severe infections from two hospitals covering a population of approximately 900,000 people. This benchmark includes multivariate algorithms that can integrate covariates describing more details of the target time series using different information available for individual admissions. The first aim of this study is to compare the performance of machine learning models to naïve seasonal models. The second aim is to investigate if the modeling error can be decreased by multivariate forecasting in which additional time series from the laboratory component and age groups are integrated. The integration of laboratory data into forecasting influenza-like illness cases has previously been approached by Pei et al*.*, showing that a Markov Chain Monte Carlo approach can be used for such a task (26). However, that study describes a benchmark based on the SIR compartmental model. It does not cover a comprehensive benchmark, including advanced multivariate machine learning algorithms for forecasting. We further investigated season-to-season variation within the laboratory component as a potential challenge for machine learning algorithms to integrate such data. We then investigated if trend forecasts with a lower temporal resolution can be used to achieve lower model errors and more reliable confidence intervals. Finally, we discuss how well forecasts reflect the true data as an outlook for its application scenarios.

## Methods

In this section, we provide further descriptions of the SARI surveillance dataset and the preprocessing procedures applied. We then describe the machine learning experiments and concepts relevant to the model validation. The results from the multivariate forecasting analyses show that the algorithms can leverage information from the laboratory component to improve the model performance. As we could not see consistency in this improvement, we applied a correlation analysis for the laboratory component to investigate the variability in these time series, which we also introduced within this section.

### Software

Data preprocessing, correlation analyses, machine learning experiments, and evaluations have been implemented using the programming language Python. We chose the time series forecasting library *AutoGluon-TS 1.1.0* as it, to the best of our knowledge, is the most recent library offering automatic machine learning, probabilistic forecasting, and a comprehensive collection of state-of-the-art univariate and multivariate forecasting algorithms, including a recent generative pre-trained transformer [11]. We used a high-performance cluster because applying all algorithms within a one-day walk-forward validation strategy, as described below, can be computationally demanding. Therefore, all forecasting experiments were performed with the support of *NeSI*, New Zealand’s eScience Infrastructure high-performance computing facilities.

### Dataset and data preprocessing

The SARI patient admissions are available at daily resolution. For consenting patients, a laboratory test was done revealing the infection with one of nine respiratory viruses covering influenza (FLU), respiratory syncytial virus (RSV), rhinovirus (RV), parainfluenza virus 1–3 (PIV), human metapneumovirus (HMPV), enterovirus (ENTV), and adenovirus (ADV). The samples used for the RT-PCR protocols are nasopharyngeal swabs and nasopharyngeal aspirates for adult and older children and for young peadiatric patients, respectively. The percentage of patients tested for these viruses varies between 60 and 100% (Fig. S1, see supplementary material). The SARI case counts, and the results from the laboratory testing are collected in a hospital's internal databases at a daily resolution and made available by the end of the day. In other words, the surveillance data is available in near real-time, which is an ideal situation for using these data as input for machine learning algorithms to train a model as soon as the data can be gathered from the database to provide an updated forecast of SARI hospitalizations for the next days or weeks. Besides the laboratory component, we created additional time series describing SARI cases that were split into different age groups. Note that our statements about real-time data availability yet describe the technical possibility of using this data as input for models applied within an information system in the future. For this study, real-time data retrieval could not be implemented due to data-sharing policies that require further approval.

In the dataset, SARI cases are recorded daily, allowing us to provide forecasts of total SARI cases at this resolution. In all forecasting experiments, the machine learning models use a time series describing the total SARI incidence as a target. The incidence is defined as *SARI per 100,000 population* using the total population of the regions serviced by the two hospitals where the SARI surveillance is implemented. For the multivariate forecasting experiments, we integrate the laboratory component describing which respiratory viruses circulate among SARI patients, and the SARI case counts split into different age groups. The virus circulation is defined as the number of positive tests for a certain virus divided by the number of patients for which a PCR test has been done, as this can vary depending on the SARI cases on a specific day and the number of consenting patients. Severe acute respiratory infection cases for specific age groups were normalized as cases per 100,000 population using the accumulated population sizes for the range of ages within the age groups of < 1 year, 1–4 years, 5–14 years, 15–64 years, and > 65 years. The age groups were defined by medical experts according to their experience with severe hospitalization risk in relation to the age of the patients.

The SARI admission numbers vary widely from day to day, especially on weekends and holidays. As we are interested in the predicted trend for the forecasting horizon rather than an exact value for a particular day in the forecast, we applied smoothing to the time series to turn the daily case counts into a trend of SARI cases. To avoid leakage of future values, we use a sliding seven-day window to compute the average of the SARI incidence of a particular day and the incidences of the previous six days (Fig. S2, see supplementary material). In this way, we maintain the daily resolution, and the window size of seven days ensures that one weekend is always covered to mitigate the potential impact of lower hospital admissions on weekends.

This smoothing strategy has been applied to the total SARI incidence and all covariates. For more details about the incidence split into different age groups and the positive test rates of the different viruses, we refer to Fig. S3 and Fig. S4, respectively (see supplementary material).

### Forecasting benchmark experiments

Forecasting models within this analysis aim to provide 21 predictions describing a 21-day forecast for the total SARI incidence. In univariate forecasting experiments, the algorithm uses only the target time series describing the total SARI incidences to train a forecasting model. In multivariate forecasting, the selected algorithms are provided with additional time series describing either the SARI cases split into different age groups or respiratory viruses. These additional time series are called *covariates* or *past covariates*. The age groups and laboratory components provide five and nine covariates, respectively. These can also be integrated simultaneously, resulting in 14 covariates integrated into the multivariate model.

The machine learning benchmark covers algorithms of various types using a collection of algorithms from the “best quality” preset as suggested by the AutoGluon library [11] covering statistical and machine learning algorithms ARIMA, ETS, Theta, CrostonSBA, and NPTS, as well as the artificial neural network algorithms PatchTST, DeepAR, and the Temporal Fusion Transformer (TFT) [27–34]. As some algorithms in the benchmark could potentially benefit from parameter tuning, we applied a grid search that systematically iterates over a grid of parameter settings specific to each algorithm and provides several forecasts from models trained using these different settings. Given an algorithm, all parameter settings are evaluated based on training-validation splits on past data from the time series to determine which setting to use for computing a forecast evaluated using the testing set. The grid size is algorithm-specific (Table S1, see supplementary material) and was determined to find a balance between comprehensiveness and computational runtime. This grid search required more than 70,000 CPU hours on the high-performance cluster. While parameter tuning explores the potential of using one algorithm with different settings, it might also be beneficial to use several algorithms in an ensemble and make model selections based on the different algorithmic concepts. To explore the potential of this strategy, we applied the AutoML. Note that AutoML was used with default settings and without parameter tuning, as the corresponding function does not provide an efficient way of applying a fine-grained walk-forward validation. However, this AutoML analysis has been included to explore its potential compared to running the algorithms in isolation.

The algorithms TFT and DeepAR are neural network architectures providing multivariate-to-univariate and multivariate-to-multivariate forecasting, respectively. Both algorithms were applied in a univariate setting to investigate the difference in model performance using a univariate and multivariate strategy but with the same algorithmic concept.

Our dataset provides valuable data collected over several years for well-defined hospital admissions at daily resolution. Nevertheless, the dataset poses a small data-learning challenge. As the surveillance is paused during the summer, we can use the daily counts of hospital admissions of 152 days per year. Transfer learning overcomes this challenge using pre-trained models based on large datasets covering data from many domains [35]. These generative pre-trained transformers (GPT), also called *foundation models*, are trained on big datasets and designed to be adjustable to new data from other or similar domains but potentially with low sample sizes. In healthcare applications, transfer learning has been shown to be beneficial, especially for image diagnosis [36, 37]. Recently, this concept has been adapted to time series forecasting [38–40]. For the reasons just explained, we integrated *Chronos*, one of the latest freely available forecasting GPTs, in the benchmark.

**Fig. 1:**
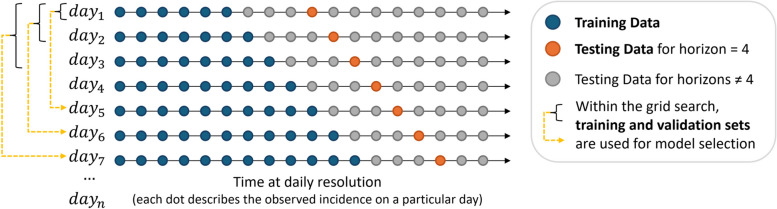
One-step walk-forward validation strategy. This scheme depicts the one-step walk-forward validation, with one day as one step. Given a particular day in the data, for example, day 7, the data is split into training and testing sets so that the training data includes this day as the last observed value (blue dots). These training samples are used to train a model that computes a forecast. The testing set (grey and orange dots) is used to evaluate the forecast. In this example, the orange dots represent the data points used to validate day 4 on the forecasting horizon. However, this strategy is applied equally for other days on the horizon. The training and testing split strategy applies when using the default parameter settings without hyperparameter tuning. For model selection, when tuning the hyperparameters, training/testing splits, created previously during the walk-forward validation, are used as training/validation splits, as indicated by the yellow dashed arrows. On day 7, for example, 3 training/validation splits can be used (day 1, day 2, day 3). The different parameter settings in the grid search are validated using these training/validation splits. As multiple training/validation splits are usually available, the average error rate defines the parameter setting with the lowest error rate (model selection). This best parameter setting is then used to train a model on the training data available for day 7. The forecast of this model is evaluated using the testing set. Model selection is skipped for the first steps in this walk-forward validation (days 1 to 4 in the figure), for which no training/validation splits are available. In this case, the default model provides the forecast. Using this strategy to create training/testing and training/validation splits within the walk-forward validation allows us to make the most use of the available data

The forecast evaluation and model selection are done within a one-day walk-forward validation (Fig. 1), reflecting the real-world scenario in which a new model should be trained after each day as soon as the most recent data for that day is available [19]. More precisely, for each day, the time series is split into the training set describing all observations until and including this day and the testing set composed of all observations after this day. As the forecasting horizon is 21 days, only the first 21 observations from the testing set are relevant to the evaluation. For the model selection, determining the best setting from the parameter grid, this validation strategy allows for using training/test splits from the past as training/validation splits. As the evaluation starts with the year 2014, no training/validation splits are available for the first forecasts in this year. In this case, no model selection is applied, and the default settings of the algorithms are used. For all other time points in the walk-forward validation, all available training/validation splits are used to compute the average performance of all parameter settings, and only the one that achieves the lowest error rate is selected. This strategy allows us to make the most use of the dataset for creating testing and validation sets from the data within the walk-forward procedure.

Epidemics of respiratory diseases show strong seasonal patterns. Therefore, making use of approximations directly derived from historical data can serve as forecasting without training a model. For this reason, we integrated two naïve baseline models within this analysis. The first one is called the *Naïve Seasonal Model*, which, given a day in the future, predicts the value for that day observed in the previous season (year). The second naïve model is the *Naïve Average Seasonal Model* that, given a day in the future, predicts the average of all values observed for this day in the previous seasons available in the historical data.

Probabilistic forecasting provides, in addition to point predictions, confidence intervals that provide a lower and upper bound of the range of values the model expects to determine for the true value based on the training data used to create the model. From this, we receive 19 probabilistic forecasts for 5% to 95% in steps of 5%. The percentage describes the expected probability that the true value will be below the value of the probabilistic forecast. Considering, for instance, the 5% forecast as the lower bound and the 95% forecast as an upper bound, the interval between these two forecasts describes the 90% confidence interval (CI). Accordingly, we created the 80% CI by using the probabilistic forecasting of 10% and 90% and the 70% CI by using the probabilistic forecasting of 15% and 85%. The quantile forecasts used to create these confidence intervals have been further evaluated using the mean pinball loss [41].

The data used for this study has gaps in the time series as the SARI surveillance has been paused from October to April in the summer months. For this reason, there are 152 daily counts of hospital admissions per year covering the time from early May to early October without any missing values for the six months the surveillance was active. On the one hand, this poses no problem as the number of severe respiratory illness cases is very low during these months, and we do not expect to experience situations that pose a challenge for public healthcare during this time. On the other hand, this results in a technical challenge as the current forecasting algorithms require the time series to be continuous without missing values [8–11, 42]. A common approach for imputation is to fill in missing values based on the last observed values, as automatically done by default when using, for instance, AutoGluon-TS [11]. However, for our dataset, this would result in a flat line between October and April for each summer, simply describing the SARI incidence last observed in October, which we found inappropriate as this could differ from year to year, but it does not provide information of any relevance to the forecasting model trained. Using forecasting to impute the large gaps is also unsuitable because data that could represent patterns during the summer months is simply not available. Therefore, we decided to fill these gaps by zero, disabling the model to learn any useful patterns from this time. Note that we completely exclude the summer months from the model evaluation for the different years.

The metric used in the evaluation is the Mean-Absolute-Percentage-Error (MAPE). We chose this metric as it is scalable and easily interpretable [9]. Given, for example, the weekly forecast of 100 cases computed by a model that achieves a MAPE of 0.1, the true value is expected to be between 90 and 110, according to the evaluation based on historical data. MAPE has been computed for the data separated by year to analyze how the forecasting error differs between different seasons. As the maximum SARI incidence can vary from year to year, we saw another advantage in using the MAPE metric as it is scale-independent, which allows for a fair year-to-year comparison. However, we also considered the Root-Mean-Squared-Error (RMSE) and Mean-Absolute-Error (MAE) to provide a comprehensive overview. For assessing how well the forecasts reflect the true SARI incidence, we also used the R-square coefficient to express the goodness of fit. Note that the first 14 days per year were excluded from the MAPE calculation to ensure that the algorithms can integrate at least 14 observations of a particular year to compute forecasts involved in the evaluation for this year.

### Correlation of lab component covariates with the SARI incidence

To further investigate the challenges of integrating the laboratory component, we performed a correlation analysis to investigate how well the time series from this data correlates with the SARI incidence, indicating how informative the information about the virus circulation could be for a SARI forecasting model. Additionally, this analysis provides further insights into the laboratory component as it could be relevant to other surveillance studies monitoring similar data types. The correlation coefficients were derived from current SARI to current virus and past virus incidences to reveal statistical relationships that could be leveraged by a machine learning algorithm to create a predictive model. Hence, for each virus, the incidence over the year was correlated with the SARI incidence over the year using the Pearson correlation. Then, we applied a temporal shift to the virus incidence values to compute correlation coefficients between the SARI incidence at a certain time point in the time series and the virus incidence observed days or weeks before this time point. These temporal shifts shall show if there are statistical patterns in the virus incidences that could be informative towards the total SARI incidence during the upcoming one, two, or three weeks.

## Results

We first summarize the results from the forecasting benchmark. These results suggest that integrating laboratory data does not consistently result in better model performance. Therefore, we describe an additional analysis based on this data before describing the impact of using forecasts at different temporal resolutions. Lastly, we evaluate the probabilistic forecasting.

### Impact of parameter tuning on the overall performance

**Fig. 2:**
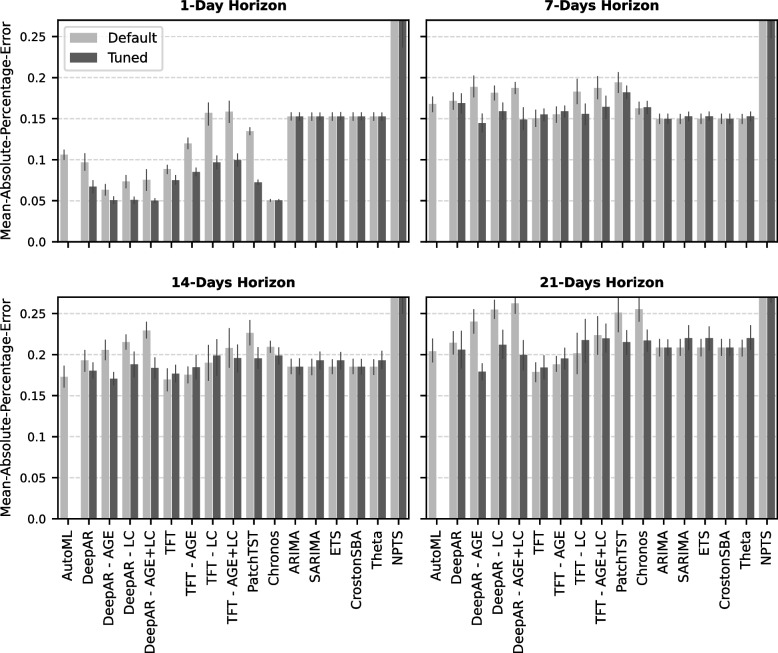
Comparing the algorithm performance with and without parameter tuning. The bars represent the mean MAPE error across all validation years with the error bar representing the 95% confidence interval. Light grey bars show the error rates for forecasts computed by models trained with default settings. The dark grey bars show the error rates achieved by models for which parameter tuning was performed by selecting a model from a grid search

Some algorithms, such as the TFT, did not benefit from parameter tuning, except for the 1-day forecast, which is the least challenging task (Fig. 2). However, DeepAR shows much lower error rates with tuned parameters and slightly improves when using the age groups, as shown for the 7-day horizon evaluation. Here, we conclude that the untuned TFT and the multivariate DeepAR using the age groups consistently achieve overall low error rates for the horizons 7 days, 14 days, and 21 days. These observations do not significantly change when using the other error metrics we explored (see Table S2 in supplementary material).

### Benchmark of forecasting algorithms within the different validation years

**Fig. 3:**
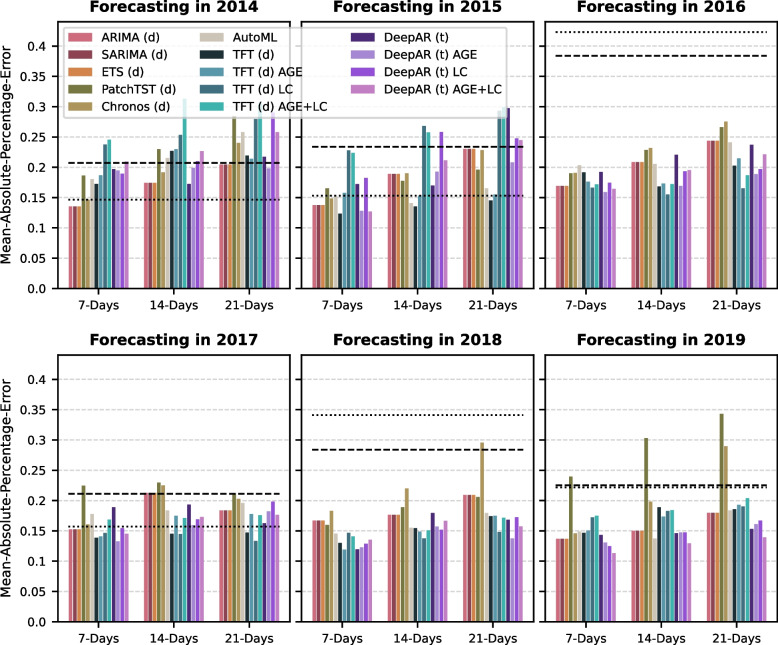
Forecasting benchmark for different horizons separated by year. The model evaluation has been split by year, represented by six sub-panels as indicated by the subfigure titles. The dashed and dotted lines show the model error of the Naïve Seasonal Model and the Naïve Average Seasonal Model, respectively. Predictions are available for each of the 21 days on the forecasting horizon. For simplicity, the plots visualize the errors only for three different time points: the maximum (21 days) and 2 intermediate time points (7 and 14 days). DeepAR and the Temporal Fusion Transformer (TFT) have also been applied for multivariate forecasting experiments. These include covariates describing the age groups (AGE), laboratory component (LC), or both (AGE + LC)

Considering the forecasting models for the 7-day forecast and beyond, the naïve models are outperformed by most of the machine learning approaches, except for the year 2014, with the univariate TFT algorithm achieving the most accurate forecasts overall (Fig. 3). As we did not see big changes in the MAPE for forecasts of slightly different days on the horizon, we here report the results of three forecasts (7 days, 14 days, and 21 days). Interestingly, the TFT algorithm did not consistently provide better models when integrating covariates from the laboratory component or age groups. In some years, the model error even increased for the multivariate TFT. In contrast, DeepAR consistently achieves low error rates in the multivariate setting, incorporating the age groups. This is observed especially in 2017, 2018, and 2019, in which the univariate TFT performes similarly. The training sets for those years are larger compared to the other years, which indicates that these neural network learners benefit from larger training sets. For the following analyses we therefore decided to explore the univariate, default TFT forecasts and the forecasts from the tuned, multivariate DeepAR using the age groups.

### Correlation of lab component covariates with the SARI incidence

**Fig. 4:**
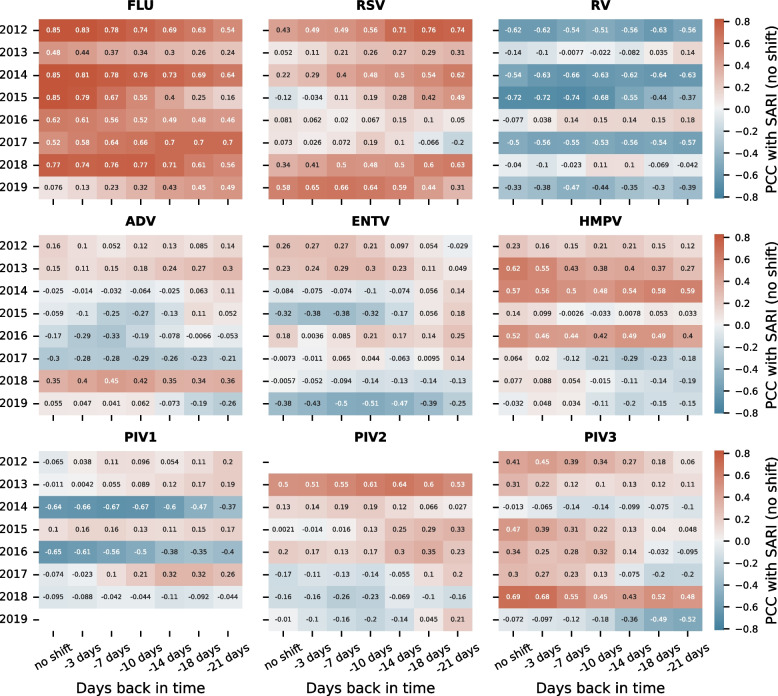
Pearson Correlation between current SARI incidence values and past incidence of detected respiratory viruses. Heatmaps show the Pearson Correlation Coefficient (PCC) between the SARI incidence and the incidence of individual respiratory viruses. The virus incidence values were shifted back in time to visualize how well past values from the different viruses correlate with present SARI values. For example, the RV value at position (-3 days, 2017) represents the Pearson correlation between the SARI incidence on a specific day and the RV incidence 3 days before that day, calculated over all time points from 2017. White areas (see PIV2 for 2012) describe cases in which the numbers were too sparse to compute a PCC. The viruses investigated are influenza (FLU), respiratory syncytial virus (RSV), rhinovirus (RV), parainfluenza virus 1–3 (PIV), human metapneumovirus (HMPV), enterovirus (ENTV), and adenovirus (ADV)

The correlation between SARI and virus incidences varies strongly from virus to virus (Fig. 4). While influenza, for example, shows a highly positive correlation, rhinovirus correlates negatively with SARI, which indicates that rhinovirus circulates before the total SARI admissions increase. For these two examples, we observe high absolute correlation coefficients. Furthermore, these can be high even if the virus incidence was taken from past observations relative to the total SARI incidence. A machine learning algorithm could potentially leverage such correlation patterns to create a predictive model. However, the correlation analysis also shows that these patterns are inconsistent; see 2013, 2016, and 2018 for rhinovirus and 2013, 2016, 2017, and 2019 for influenza.

### Forecasting resolution and probabilistic forecasts

**Fig. 5:**
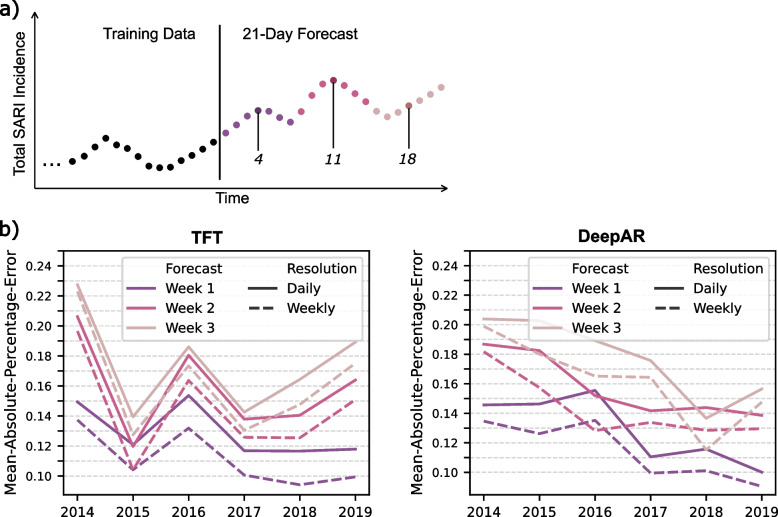
Forecasting error of predictions at two different resolutions. **a** Visualization of how the forecasting output is changed from daily to weekly resolution. The black dots represent the last observations in the time series used within the training set. The vertical black line represents the time point at which the time series has been split into training and testing sets, with 21 dots on the right-hand side representing the predictions for 21 days. The forecasting at daily resolution represents 21 values for 21 days. We analyzed the impact of changing the resolution by averaging over 7 days, as indicated by different colors, to compute a trend for the upcoming three weeks rather than 21-point predictions for each day. There are three mid-week time points at 4, 11, and 18 days. The forecasts for these days are used to compare the single-point forecast of a day (daily resolution) to the average of the forecasts for the week (weekly resolution). **b** The lines represent the model error for different years when the forecasting is used at different temporal resolutions as described in **a**

The incidence values within the dataset are available at daily resolution. The algorithms used to train forecasting models aim to capture statistical patterns in this data and provide forecasts at the same temporal resolution. However, a daily forecast is not necessarily required, depending on how the forecasting is used. In hospitals, for example, it might be sufficient to consider weekly trends for proactive planning instead of considering the forecast of every single day on the forecasting horizon. Therefore, we explore if the model error can be reduced when changing the forecasting resolution. Instead of using 21 predictions for 21 days, we use three predictions representing the forecasted trend for the upcoming three weeks. This resolution change is done by using the average forecast of the days within the first, second, and third weeks (Fig. 5a).

The expectation is that the resulting trend prediction better captures the time series trend compared to the forecast at daily resolution for hospital admissions, which can vary strongly from day to day. For example, forecasting at weekly resolution will be sufficient to foresee rises in the healthcare burden, and noise in the daily forecast could potentially be mitigated by using a weekly trend represented by the average of several daily forecasts. Indeed, using the forecasting at weekly resolution and comparing these to the weekly averaged SARI incidence in the testing data results in lower model errors compared to using the data at daily resolution (Fig. 5b).

**Fig. 6:**
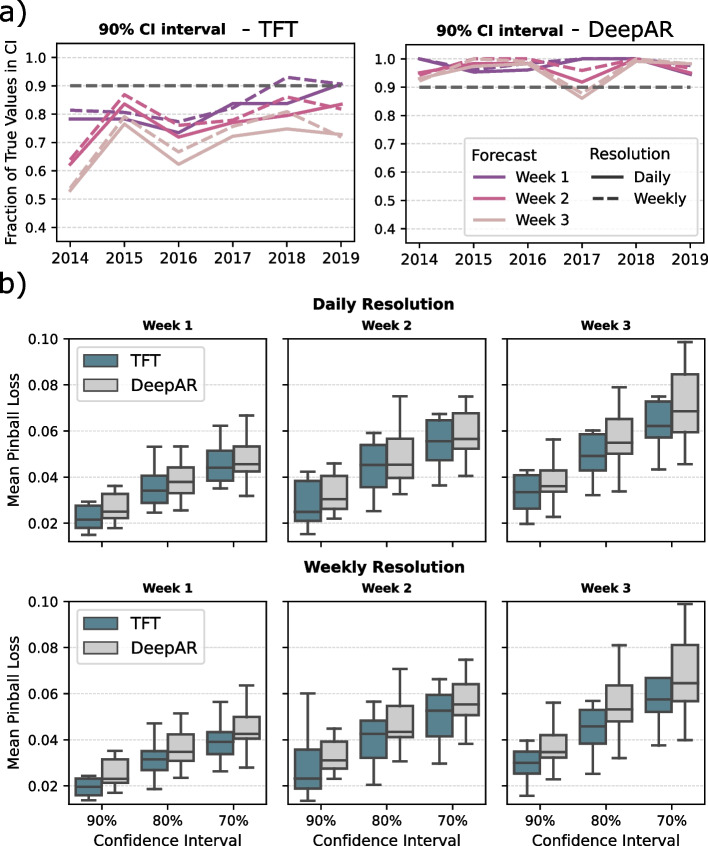
Evaluation of probabilistic forecasting. **a** The 90% confidence interval is validated by the fraction of true values captured within the confidence interval during the one-day walk-forward validation. The dashed black line represents the expected probability of the true value lying within the confidence interval estimated by the model. The colored lines represent the actual fraction of values from the testing sets that are within the confidence interval when applying the one-day walk-forward validation. Again, the forecasts are evaluated at different temporal resolutions. **b** The quantile levels are validated for the different confidence intervals 70%, 80%, and 90% using the mean pinball loss. This has been done for the two algorithms in comparison, as indicated by the colors. Subtitles describe the horizon and forecast resolution

Probabilistic forecasting can be used to derive a confidence interval for a point prediction from the model. However, when applying the one-day walk-forward validation, the model's confidence about observing the true value within a certain range of values does not necessarily reflect the actual fraction of true values lying within the confidence intervals. Therefore, we use the testing sets from the one-day walk-forward validation to compute the fraction of true values within the confidence intervals and compare it to the probability of observing the true value within this interval as estimated by the algorithm. In most of the cases, this fraction does not precisely agree with the estimated probability, suggesting that the probabilistic forecasts are moderately accurate (Fig. 6a).

It is visible from this analysis that the TFT computes quantiles that result in smaller confidence intervals, and the fraction of true observations within the confidence intervals is lower than the expected probability that the true value lies within the confidence interval. In contrast, the quintile forecasts from DeepAR result in much larger confidence intervals. This leads to a very high fraction of true observations within the confidence intervals (Fig. 6a) but less useful confidence intervals as they provide no clear orientation about where to expect the true observation. Therefore, we conclude that the TFT provides confidence intervals more suitable for practical application. Overall, the TFT also achieves lower pinball loss scores computed for the quantile levels of the different confidence intervals (Fig. 6b), which confirms the conclusion drawn from the previous analysis.

## Discussion

The benchmark analysis concludes that the Temporal Fusion Transformer (TFT) and DeepAR achieve the overall lowest forecasting error, especially for the one-week and two-week hospital admissions forecasts (Fig. 2, Fig. 3). While the TFT performs well with default settings and without using any covariates, DeepAR substantially benefits from parameter tuning and slightly improves the forecasting performance by integrating the SARI age groups. Additional analyses describe that DeepAR slightly outperforms TFT when evaluating the point forecast (Fig. 5). However, these improvements of DeepAR over TFT are extremely small, whereas the confidence intervals provided by TFT are much more reliable than those computed by DeepAR (Fig. 6).

Chronos could not leverage its potential of using a large pre-trained transformer to apply transfer learning, which agrees with findings from other forecasting-related studies concluding that transfer learning does not always result in the best-performing model [43]. Remarkably, Chronos performed very well on the 1-day and 7-day horizon for 2014 (Table S2, see supplementary material), for which the training data was the smallest in our analysis, posing the biggest potential for transfer learning when applied to small training datasets [35].

Interestingly, the AutoML approach, which includes the TFT in the algorithm collection, could not provide better forecasts in comparison to using TFT separately. This indicates overfitting of the internal validation of AutoML. In other words, the model weights AutoML creates based on the internal validation result in a weighted ensemble forecasting that is less generalizable to the testing data. One reason for this could be that AutoML considers the average model error over all time points on the horizon. As we see large differences in the forecasting error for days early and late on the horizon, we suggest considering the model errors for different time points on the horizon to compute weights individually. Additionally, it could be beneficial to integrate the stepwise walk-forward validation within the AutoML function to avoid redundant computing and make parameter tuning more efficient for AutoML within such a comprehensive validation. However, this would require substantial changes to the AutoML library we used, which we see as beyond the scope of this study.

None of the multivariate algorithms in the benchmark could leverage information from the laboratory data to improve the forecasts consistently (Fig. 2 and Fig. 3). On the one hand, this is surprising because information about the virus circulation could be relevant to a model forecasting how SARI admissions behave in the upcoming weeks. On the other hand, it is known that the incidences of respiratory viruses and their correlation with the SARI incidence strongly vary from season to season, which we could also observe in our data (Fig. 4). Such strong variations pose a challenge for machine learning algorithms aiming to leverage consistent and generalizable patterns in the data. In other machine learning-based studies, it has also been shown that cases exist in which multivariate forecasting achieves only small improvements over univariate forecasting [44], as we observe for DeepAR integrating the age groups, or that the multivariate approaches even increase the error rate in comparison to the univariate approaches [45]. Considering the age groups, the dynamics in the < 1 year age group provide predictive information about increases in the SARI incidence (Fig. S3, see supplementary material). As this is moderately consistent, DeepAR could leverage this information to decrease forecasting errors slightly (Fig. 2). Such predictive dynamics are visible in some years for viruses, such as RSV or rhinovirus, but in contrast to the < 1 year covariate, these dynamics in the laboratory component vary from year to year and are not present in all years (Fig. S4, see supplementary material).

In general, this dataset is based on low admission counts as it describes severe cases of respiratory illness requiring inpatient hospitalization. This causes strong day-to-day fluctuations in the admissions, which required the 7-day sliding window smoothing (Fig. S2, see supplementary material). While the overall coverage of laboratory tests is high (Fig. S1, see supplementary material), the SARI cases split into nine different viruses result in sparse time series that could be too sparse to properly reflect the infectious dynamics caused by the different viruses, potentially challenging the multivariate algorithms.

**Fig. 7:**
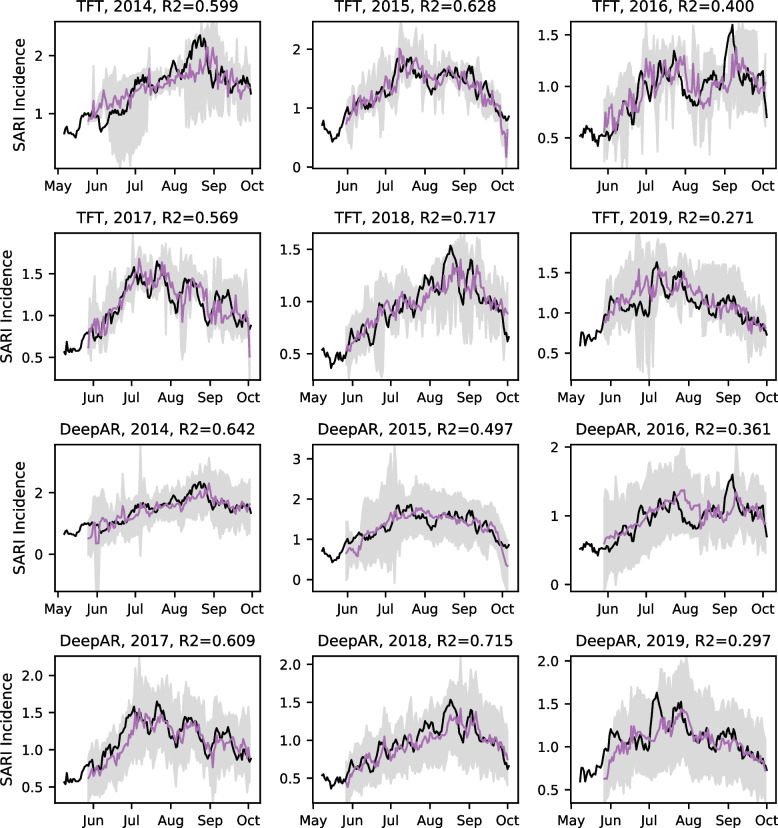
Point and probabilistic forecast of the Week 1 forecast in comparison to the true SARI incidence. Forecasts were extracted from the 1-day walk-forward validation, and the temporal resolution for the point and probabilistic forecasts were changed from daily to weekly. The purple line represents the forecast, and the shaded area is the 90% confidence interval derived from the probabilistic forecasting estimated during the model training. The black line shows the truly observed SARI incidence after applying the 7-day sliding window smoothing. The subpanel titles describe the algorithm and the validation year. These titles also show the R-square scorer (R2) describing the goodness of fit of the forecast

While the SARI surveillance captures the seasonal epidemics of severe respiratory disease cases during the winter months, the dynamics during each season, described by the strength and timing of surges, strongly vary from season to season (Fig. 7). This might explain why SARIMA, expecting a seasonality of one year, shows no improvement over ARIMA.

In the practical application of SARI forecasts in hospital management, using forecasts at daily resolution might be unnecessary. The daily forecast could include noise that makes forecasts inaccurate, and such noise can be mitigated by using average values from the forecast, which comes at the cost of decreasing the temporal resolution. We analyzed the impact of using average weekly forecasts on the model error and reliability of the probabilistic forecasts. In both evaluations, the performance improved when using weekly forecasts (Fig. 5 and Fig. 6).

Regarding the probabilistic forecasting, we could show that the confidence interval estimated by the model does not match the actual fraction of true SARI incidence values from the testing sets within the confidence interval in the one-day walk-forward validation. In most cases, the fraction of true values observed within the confidence interval was lower than the expected probability, especially for the Week 2 and Week 3 forecasts using the TFT. The confidence intervals can generally be large, making it impossible to foresee whether the SARI incidence will increase or decrease. We observe this problem, especially for the confidence intervals computed by DeepAR (Fig. 7). In conclusion, the results from this evaluation suggest that the TFT provides more reliable confidence intervals but that the actual confidence of the model is about 10% lower than estimated by the algorithm during the model training. The mean pinball loss metric reflects this advantage of TFT over DeepAR. From these analyses, we conclude that an evaluation of the probabilistic forecasts is highly important to future studies in which model confidence is relevant.

Interestingly, the point forecasts of DeepAR and the probabilistic forecasts of TFT are better for 2018 and 2019, in which the training data was much larger compared to 2014 and 2016. The statistical models, such as ARIMA, SARIMA, and ETS, performed better in the cases with low sample size, indicating that the neural networks are more effective in leveraging information from larger training sets.

To assess the forecasting for its practical application, we compare the one-week forecasts from TFT and DeepAR models and the true SARI incidence for the years used within the evaluation (Fig. 7). The true values mostly lie within the 90% confidence interval, which agrees with the previous evaluations (Fig. 6). The point forecasts can be close to the true SARI incidence shown for 2015 and 2017. However, it can fail to predict peaks during the winter season, as shown for the mid-August peaks in 2014 and 2018. More such errors are observed for the two-week and three-week forecasts (Fig. S5 and Fig. S6, see supplementary material). Remarkably, the confidence intervals are large for most of the time periods in which the point forecast is inaccurate. This is important as it indicates that large confidence intervals can be used to anticipate inaccurate forecasts during decision-making.

Regarding the practical application of SARI forecasts in Auckland, we conclude that the one-week predictions are useful as orientation about the trend prediction for the upcoming seven days, which allows for considering the forecast to make short-term decisions that can be relevant to anticipated staffing or the schedule of elective surgeries. The R-square scores achieved for this forecasting horizon are all positive and describe moderate goodness of fit of the forecasts (Fig. 7). This is different for the two-week and three-week forecasts, for which the predictions are less accurate, showing negative R-square scores for some cases, which indicates that these forecasts are too inaccurate for practical application (Fig. S5 and Fig. S6, see supplementary material). Machine learning models achieve much lower error rates in comparison to naïve strategies solely based on seasonality for most of the evaluation years, emphasizing the relevance of such models for predicting healthcare burden. Furthermore, the machine learning algorithms are flexible and can be extended in the future, potentially improving the forecasting models. It will, for example, be relevant to integrate information about the location of the hospital, such as intensive care and high-dependency units in these locations. In contrast to the models for total SARI forecasting, as investigated in this study, specialized models for the two different hospitals in Auckland City, Central Business District, and Counties Manukau, South Auckland, might be more accurate. Using time series specific to the hospitals will result in fewer SARI cases to be used for model training, which might pose a challenge for the algorithms related to data sparsity. However, in comparison to statistical forecasting models, machine learning is expected to provide more reliable models when data is sparse. We see great potential in using such models to compute forecasts, which will probably improve when the algorithms are provided with data specific to the hospital locations, as differences in socioeconomic characteristics can have an impact on the dynamics of disease outbreaks [46, 47]. Furthermore, such data can be integrated into multivariate forecasting, allowing algorithms to capture predictive patterns across hospital locations, which can further improve the models. As the data needed to perform this analysis needs linkage datasets that are not yet available, we leave this for future work.

## Conclusion

In this study, we explored the potential of forecasting models to predict the healthcare burden, in particular hospital admissions, caused by severe influenza-like illness. Machine learning algorithms achieve much better results in comparison to naïve seasonal approaches, emphasizing the relevance of using such algorithms in hospital management in Auckland, New Zealand. The evaluation of the probabilistic forecasting suggests that such evaluations are extremely important as the estimated confidence intervals may differ when validated on test datasets. We use strategies to change the temporal resolution of forecasts, which shows that it can be better to use forecasts at lower resolution as these can result in better trend forecasting.

The relevance of this study is underlined by the COVID-19 pandemic. While this study investigates forecasting models for seasonal epidemics without external influence through social distancing policies, it serves as the groundwork for future studies tackling the challenges arising from concept drifts in the data caused by lockdowns and strict border closure policies imposed in New Zealand in the years 2020 and 2021. Furthermore, by the end of the year 2024, the data for three early post-pandemic years will be available. This data and the results from this study will play a crucial role in future research investigating the challenges of integrating data recorded during the pandemic when modeling influenza-like illness hospitalizations in the future.

## Supplementary Information

Supplementary Material 1.

## Data Availability

The data used for this study describes sensitive information about the hospitalization of patients and their laboratory results. These can be published in the form of visualizations, which we use in the manuscript. However, the raw data about SARI hospitalizations in Auckland, New Zealand, cannot be made publicly available due to the sensitivity of the patient data. We share the analysis scripts and forecasting results within this repository: https://github.com/salbrec/SFS.git.
