# Supplementary material for: Forecasting severe respiratory disease hospitalizations using machine learning algorithms
Source: BMC Med Inform Decis Mak. 2024 Oct 9;24:293. doi: 10.1186/s12911-024-02702-0 (PMC11462891; doi:10.1186/s12911-024-02702-0)
Supplement: Supplementary file 1 — Supplementary Material 1. [file 12911_2024_2702_MOESM1_ESM.pdf]

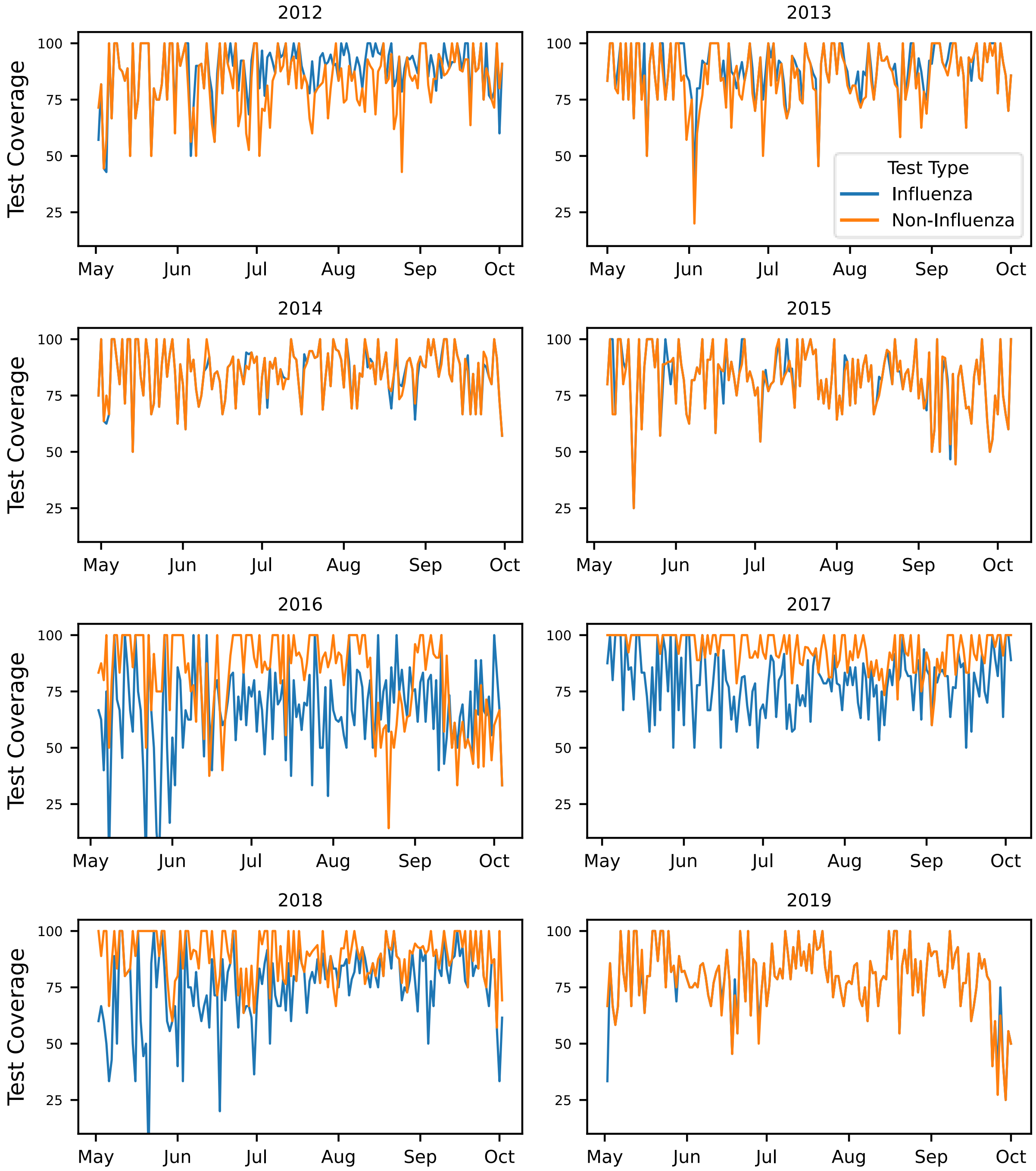

Figure S1 – Percentage of SARI cases tested for influenza or other viruses

Percentage of SARI patients tested for influenza (blue line) or other viruses (orange line).

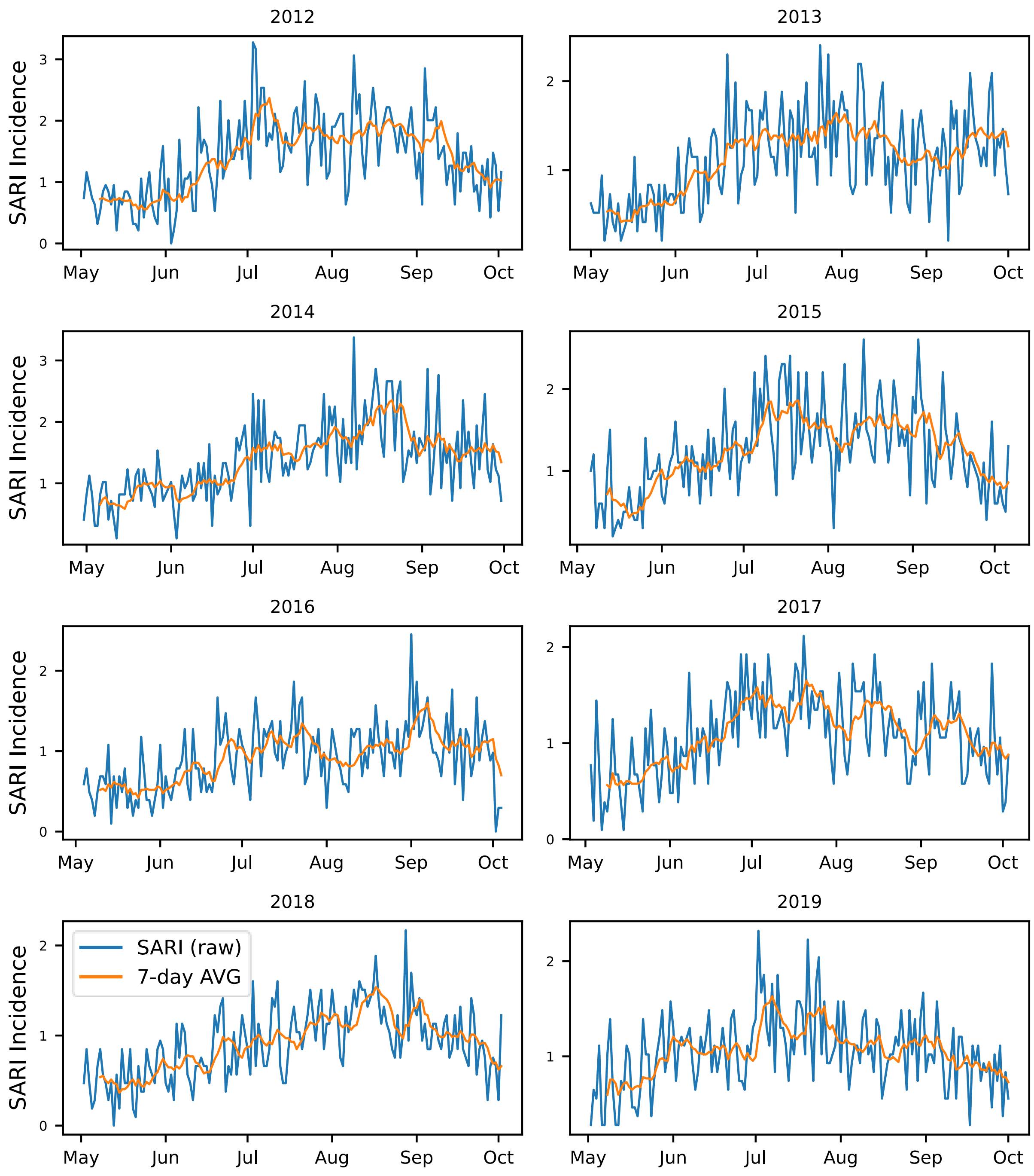

Figure S2 – Raw SARI incidence and the 7-day sliding window average SARI

The blue line shows the raw SARI incidence at daily resolution. The SARI incidence describes hospitalizations categorized as SARI per 100,000 people based on the population of the districts covered by the two hospitals where the SARI surveillance has been implemented. Due to strong fluctuations, especially on weekend days, we decided to use a smoothed SARI incidence calculated as the average of the SARI incidence over the past 7 days (orange line).

a) Incidence for the age group "< 1 year" (blue) and the total SARI incidence (black)

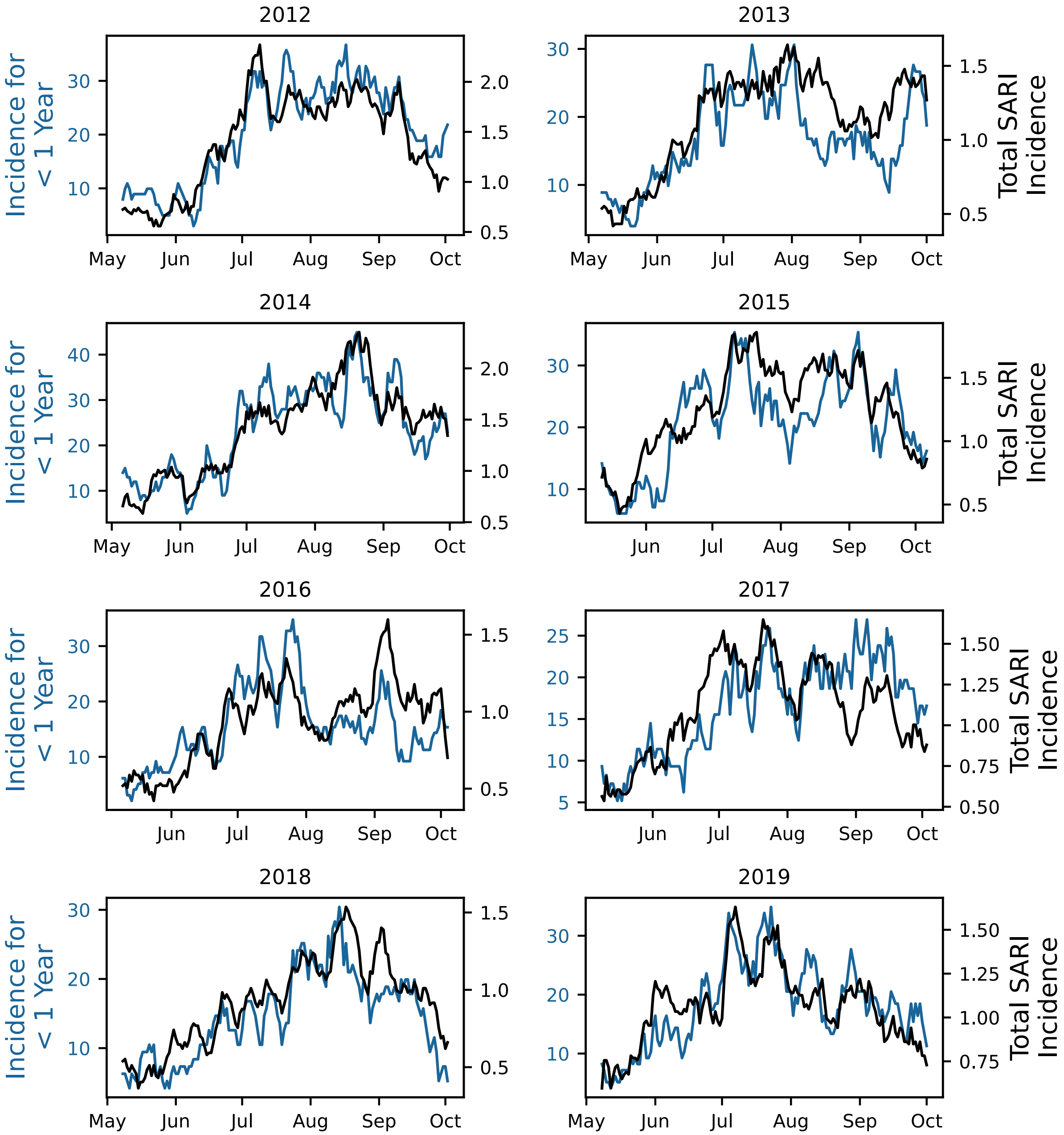

b) Incidence for the age group "1 to 4 years" (blue) and total SARI incidence (black)

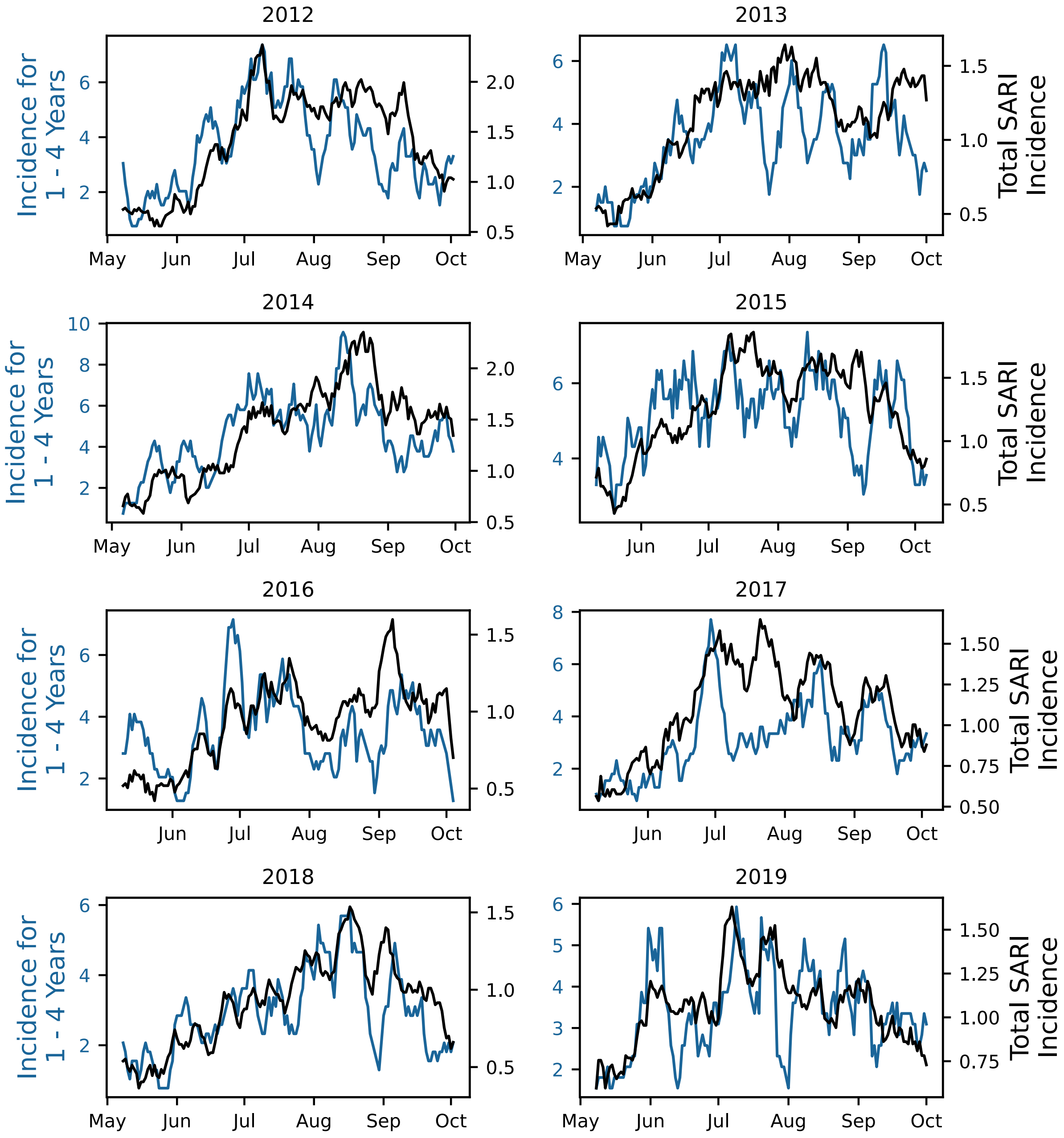

c) Incidence for the age group “5 to 14 years” (blue) and total SARI incidence (black)

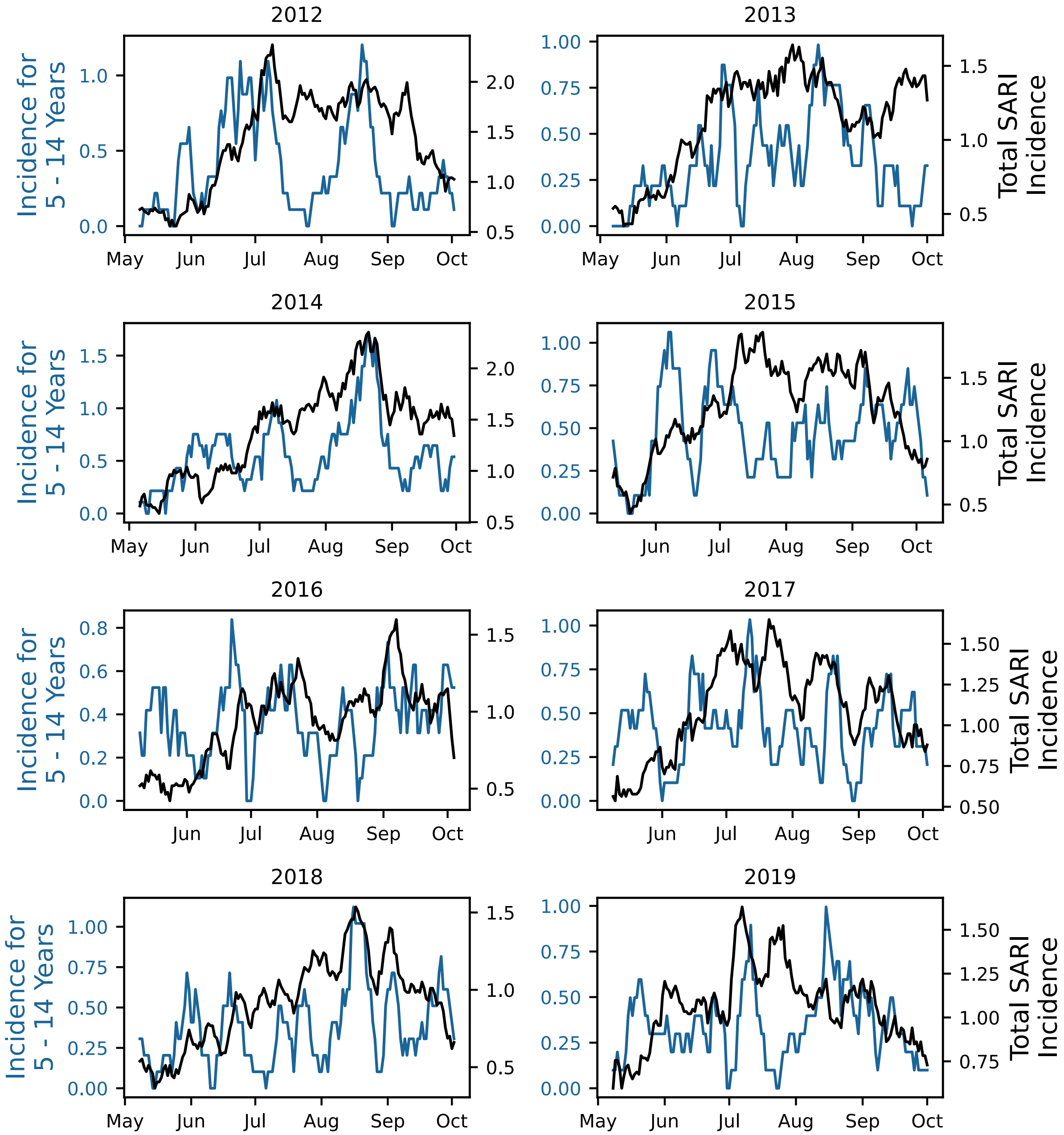

d) Incidence for the age group "15 to 64 years" (blue) and total SARI incidence (black)

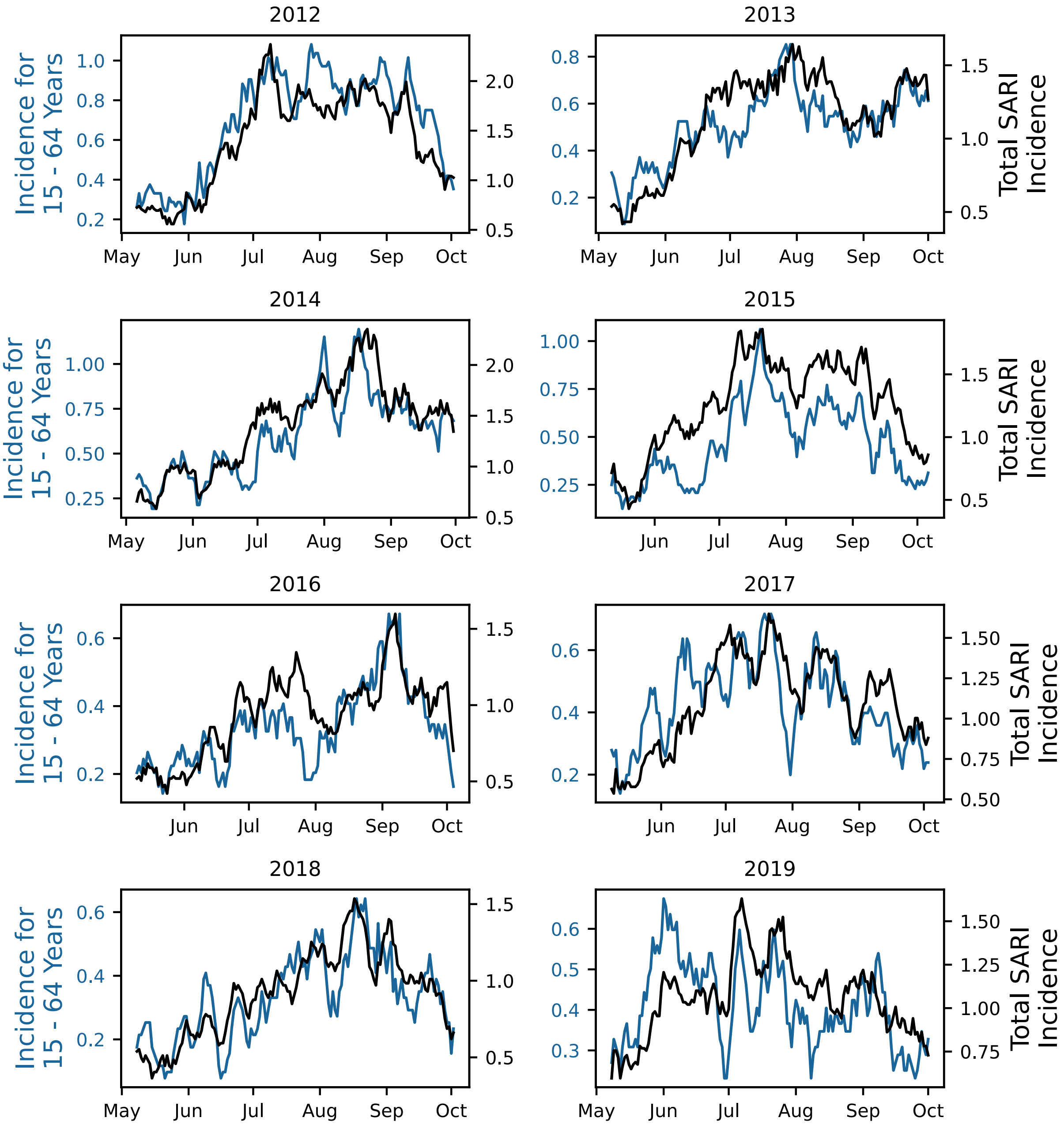

e) Incidence for the age group "65+ years" (blue) and total SARI incidence (black)

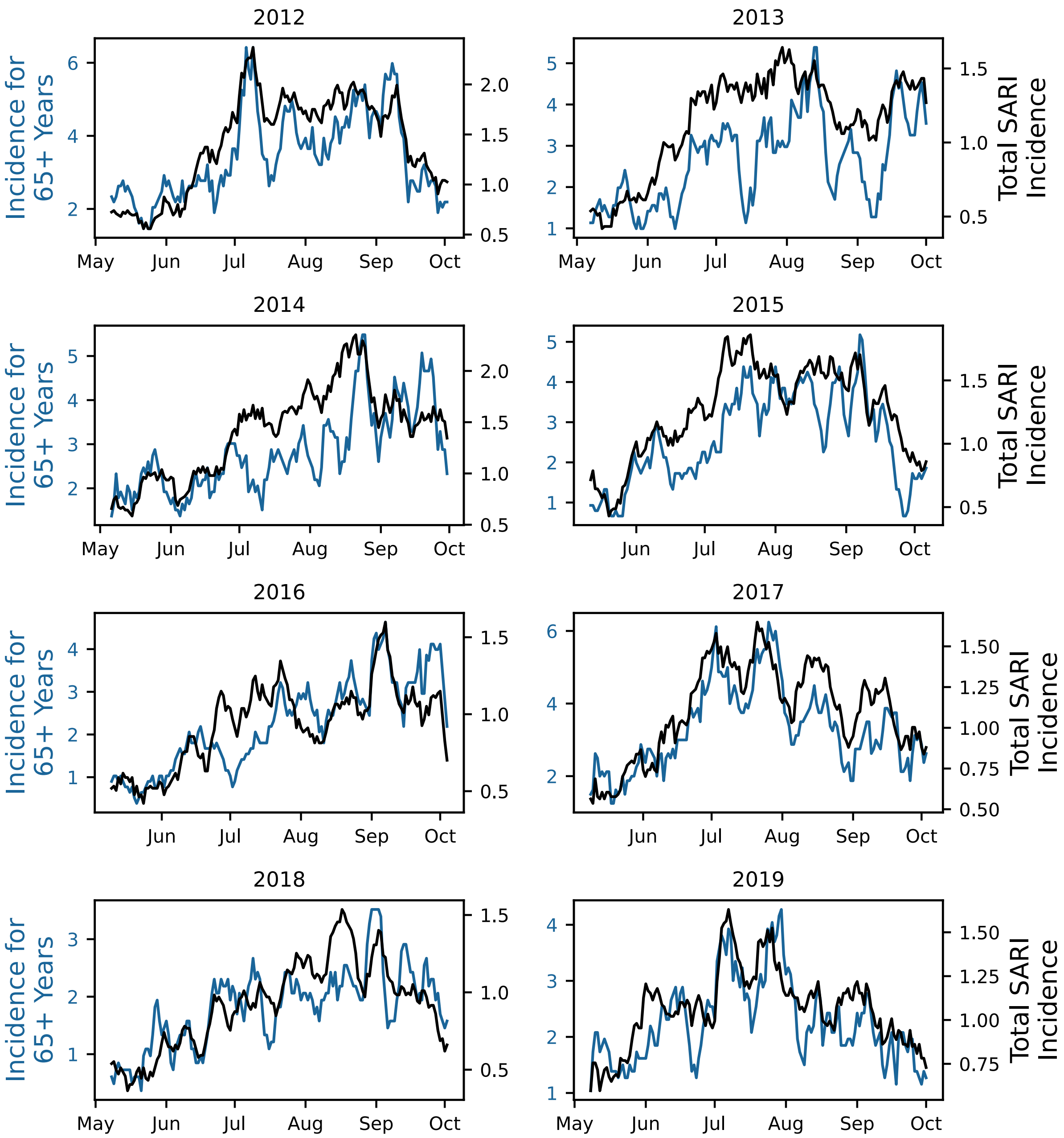

Figure S3 – Comparison between the total SARI incidence and the SARI incidence specific to different age groups as indicated by the subtitles a) to e).

The black line describes the total SARI incidence (y-scale on the right-hand side). The blue line (y-scale on the left-hand side) represents the SARI incidence by age group. The incidence always describes SARI cases per 100,000 people using the population numbers available for the different years. For the "total SARI incidence," the population size represents the number of people living in the districts covered by the two public hospitals where the SARI surveillance is implemented. For the age group-specific incidence, a subpopulation for these districts is used that represents the number of people of a certain age as specified by the corresponding age group.

a) Positive rate of influenza (FLU), respiratory syncytial virus (RSV), and rhinovirus (RV) (colored lines) in comparison to the total SARI incidence (black).

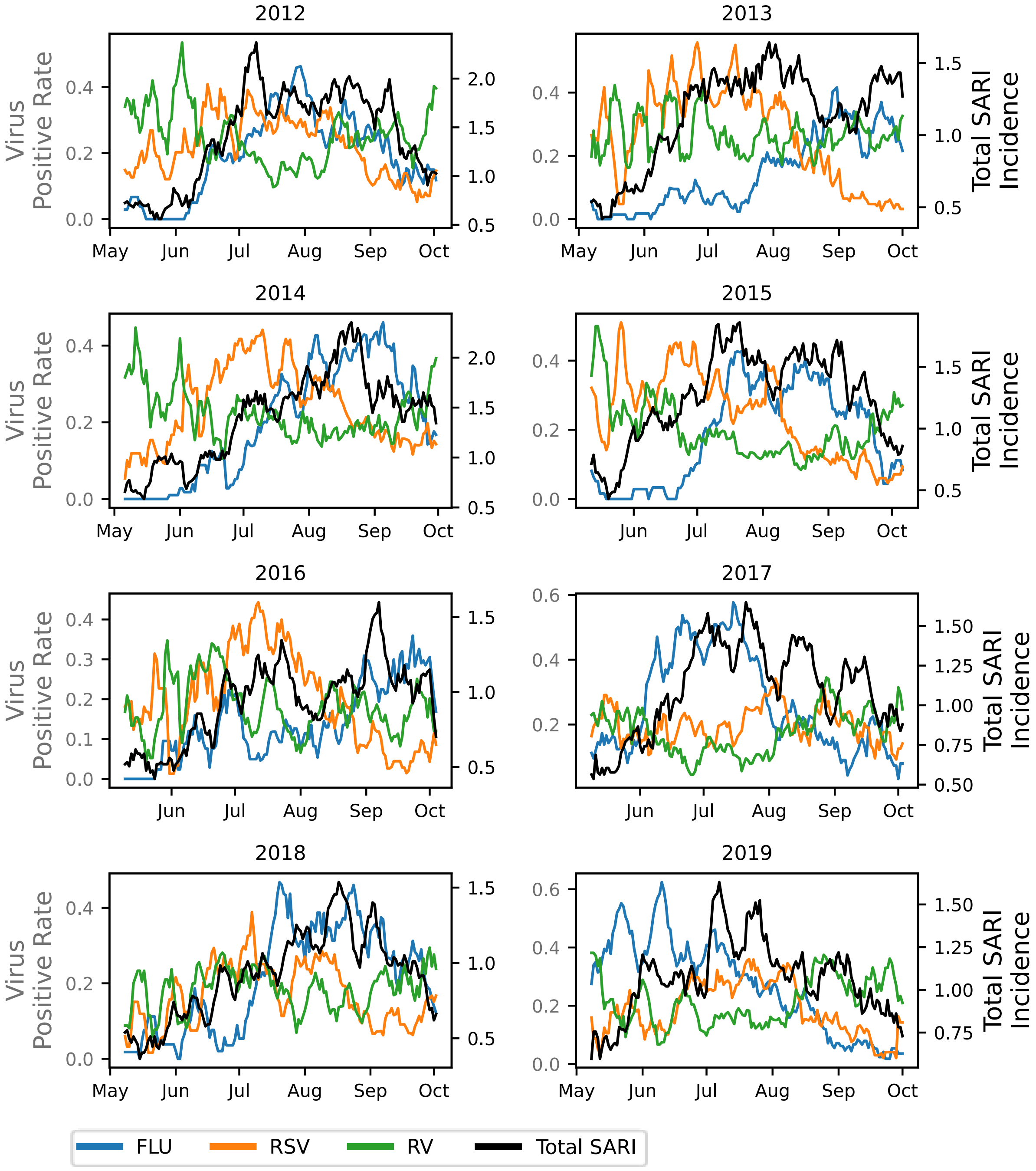

b) **Positive rate of enterovirus (ENTV), adenovirus (ADV), human metapneumovirus (HMPV), and parainfluenza virus 1-3 (PIV) in comparison to the total SARI incidence (black).**

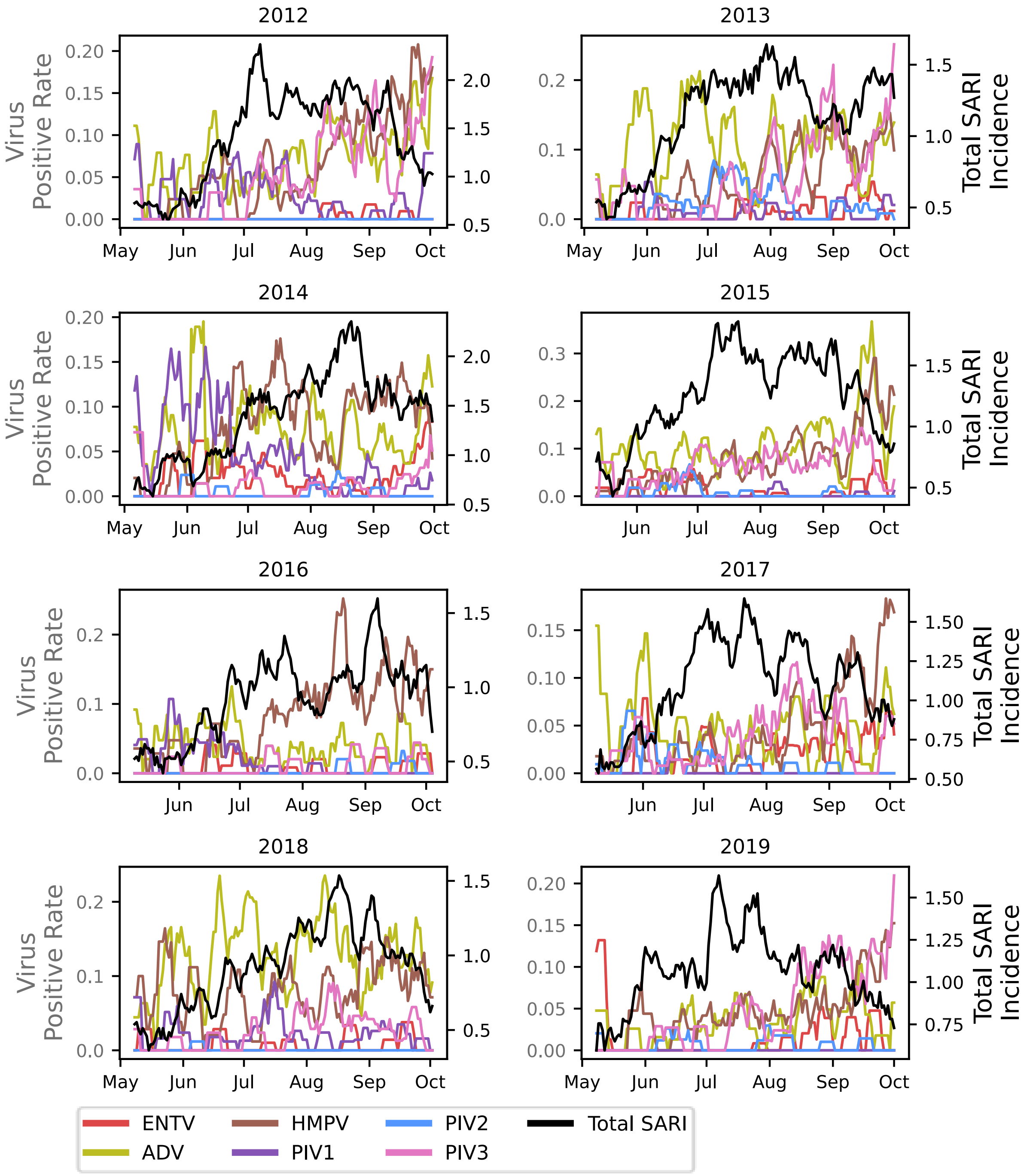

Figure S4 – Comparison between the total SARI incidence and the positive rates of different viruses derived from the laboratory component.

The black line describes the total SARI incidence (y-scale on the right-hand side). The y-scale on the left-hand side describes the rate of positive tests for different viruses, shown by the colored lines.

**TFT – Temporal Fusion Transformer** – link<br>'context_length':[*h*, 2**h*], 'hidden_dim':[32,64], 'variable_dim':[32,64], 'lr':[1e-2, 1e-3, 1e-4], 'dropout_rate':[0.1,0.2,0.5]
**DeepAR** – link<br>'context_length':[*h*, 2**h*], 'num_layers':[2, 4], 'dropout_rate':[0.1,0.2,0.5], 'hidden_size':[40, 80], 'lr':[1e-2, 1e-3, 1e-4]
**ARIMA** – using the AutoARIMA model<br>'max_p':[2**h*], 'max_q':[2**h*], 'max_d':[3], 'seasonal':[False], 'allowdrift':[True,False], 'allowmean':[True, False], 'stationary':[True,False], 'max_ts_length':[750,1500,2500]
**SARIMA** – using the AutoARIMA model<br>'max_p':[2**h*], 'max_q':[2**h*], 'max_d':[3], 'max_P':[2**h*], 'max_Q':[2**h*], 'max_D':[3], 'seasonal':[True], 'seasonal_period':[365], 'allowdrift':[True,False], 'max_ts_length':[750,1500,2500], 'allowmean':[True, False], 'stationary':[True,False]
**ETS** – using the AutoETS model<br>'model':['ZZZ'], 'seasonal_period':[None, 365], 'damped':[True, False], 'max_ts_length':[750,1500,2500]
**NPTS** – link<br>'kernel_type':['exponential', 'uniform'], 'use_seasonal_model':[True, False], 'exp_kernel_weights':[1,2,3], 'num_default_time_features':[1,2,3], 'max_ts_length':[750,1500,2500]
**CrostonSBA** – link<br>'max_ts_length':[750,1500,2500]
**Chronos** – link<br>'model_path':['large'], 'batch_size':[16, 32, 64], 'num_samples':[20,40,80], 'context_length':[None, *h*,2**h*,4**h*]
**Theta** – link<br>'decomposition_type':['multiplicative', 'additive'], 'seasonal_period':[None, 365], 'max_ts_length':[750,1500,2500]
**PatchTST** – link<br>'context_length':[*h*, 96], 'patch_len':[8, 16], 'stride':[8, 16], 'd_model':[32, 64], 'nhead':[4,8], 'lr':[1e-3, 1e-2, 1e-4], 'batch_size':[32,64]

Table S1 – Parameters explored within the grid search applied for hyperparameter tuning.

The library AutoGluon-TS provides the implementation of all algorithms used. For ARIMA and ETS, automated solutions were leveraged that automatically explore different settings for the orders (ARIMA and SARIMA) or configuration of the model components (ETS). Some parameter settings depend on the size of the horizon as indicated by the *h*, which is 21 days in all experiments.

**1-Day Horizon**											
				**2014**			**2015**			**2016**	
Algorithm	Covariates	Parameters	MAPE	MAE	RMSE	MAPE	MAE	RMSE	MAPE	MAE	RMSE
ARIMA	no	default	0.139	0.197	0.251	0.149	0.187	0.231	0.167	0.167	0.215
ARIMA	no	tuned	0.139	0.197	0.251	0.149	0.187	0.231	0.167	0.167	0.215
SARIMA	no	default	0.139	0.197	0.251	0.149	0.187	0.231	0.167	0.167	0.215
SARIMA	no	tuned	0.139	0.200	0.253	0.149	0.187	0.231	0.167	0.167	0.215
ETS	no	default	0.139	0.197	0.251	0.149	0.187	0.231	0.167	0.167	0.215
ETS	no	tuned	0.139	0.200	0.253	0.149	0.187	0.231	0.167	0.167	0.215
Theta	no	default	0.139	0.197	0.251	0.149	0.187	0.231	0.167	0.167	0.215
Theta	no	tuned	0.139	0.200	0.253	0.149	0.187	0.231	0.167	0.167	0.215
CrostonSBA	no	default	0.139	0.197	0.251	0.149	0.187	0.231	0.167	0.167	0.215
CrostonSBA	no	tuned	0.139	0.197	0.251	0.149	0.187	0.231	0.167	0.167	0.215
NPTS	no	default	0.570	0.874	0.936	0.514	0.718	0.771	0.393	0.420	0.475
NPTS	no	tuned	0.223	0.341	0.454	0.234	0.285	0.367	0.442	0.400	0.461
PatchTST	no	default	0.143	0.191	0.251	0.134	0.159	0.211	0.120	0.106	0.131
PatchTST	no	tuned	0.076	0.103	0.137	0.068	0.088	0.112	0.082	0.077	0.096
Chronos	no	default	**0.052**	**0.077**	**0.096**	**0.050**	**0.066**	**0.081**	0.055	0.053	0.068
Chronos	no	tuned	**0.053**	**0.077**	**0.092**	**0.051**	**0.066**	**0.080**	0.054	0.053	0.067
TFT	no	default	0.112	0.159	0.203	0.085	0.107	0.149	0.096	0.092	0.119
TFT	no	tuned	0.079	0.111	0.143	0.064	0.080	0.104	0.100	0.097	0.124
TFT	AGE	default	0.140	0.194	0.237	0.115	0.145	0.184	0.141	0.132	0.174
TFT	AGE	tuned	0.096	0.130	0.169	0.078	0.100	0.124	0.103	0.098	0.126
TFT	LC	default	0.200	0.254	0.310	0.194	0.261	0.331	0.168	0.158	0.214
TFT	LC	tuned	0.116	0.149	0.211	0.109	0.144	0.210	0.119	0.110	0.159
TFT	AGE+LC	default	0.189	0.252	0.319	0.207	0.275	0.368	0.151	0.142	0.194
TFT	AGE+LC	tuned	0.124	0.151	0.219	0.086	0.111	0.149	0.126	0.118	0.151
DeepAR	no	default	0.116	0.158	0.191	0.129	0.156	0.194	0.114	0.105	0.132
DeepAR	no	tuned	0.076	0.104	0.128	0.100	0.124	0.186	0.070	0.064	0.090
DeepAR	AGE	default	0.089	0.118	0.152	0.085	0.101	0.136	**0.049**	**0.047**	**0.059**
DeepAR	AGE	tuned	0.064	**0.085**	0.118	0.065	0.078	0.097	**0.047**	**0.046**	**0.058**
DeepAR	LC	default	0.107	0.138	0.195	0.087	0.102	0.140	0.067	0.060	0.072
DeepAR	LC	tuned	0.063	0.086	**0.111**	0.062	0.076	0.100	0.052	**0.048**	**0.061**
DeepAR	AGE+LC	default	0.137	0.181	0.313	0.081	0.099	0.135	0.059	0.054	0.068
DeepAR	AGE+LC	tuned	**0.062**	0.090	0.114	**0.052**	**0.069**	**0.080**	**0.050**	0.048	0.062

**1-Day Horizon**											
				**2017**			**2018**			**2019**	
Algorithm	Covariates	Parameters	MAPE	MAE	RMSE	MAPE	MAE	RMSE	MAPE	MAE	RMSE
ARIMA	no	default	0.156	0.177	0.214	0.164	0.157	0.198	0.140	0.160	0.204
ARIMA	no	tuned	0.156	0.177	0.214	0.164	0.157	0.198	0.140	0.160	0.204
SARIMA	no	default	0.156	0.177	0.214	0.164	0.157	0.198	0.140	0.160	0.204
SARIMA	no	tuned	0.156	0.177	0.214	0.164	0.157	0.198	0.140	0.160	0.204
ETS	no	default	0.156	0.177	0.214	0.164	0.157	0.198	0.140	0.160	0.204
ETS	no	tuned	0.156	0.177	0.214	0.164	0.157	0.198	0.140	0.160	0.204
Theta	no	default	0.156	0.177	0.214	0.164	0.157	0.198	0.140	0.160	0.204
Theta	no	tuned	0.156	0.177	0.214	0.164	0.157	0.198	0.140	0.160	0.204
CrostonSBA	no	default	0.156	0.177	0.214	0.164	0.157	0.198	0.140	0.160	0.204
CrostonSBA	no	tuned	0.156	0.177	0.214	0.164	0.157	0.198	0.140	0.160	0.204
NPTS	no	default	0.495	0.601	0.643	0.443	0.462	0.514	0.592	0.668	0.691
NPTS	no	tuned	0.222	0.258	0.297	0.299	0.258	0.320	0.213	0.242	0.298
PatchTST	no	default	0.136	0.142	0.189	0.123	0.104	0.128	0.150	0.158	0.202
PatchTST	no	tuned	0.078	0.081	0.105	0.064	0.058	0.077	0.067	0.073	0.086
Chronos	no	default	0.048	**0.054**	0.068	0.051	0.047	0.058	0.046	0.051	0.066
Chronos	no	tuned	0.049	0.055	0.071	0.049	0.045	0.057	0.046	0.051	0.066
TFT	no	default	0.079	0.091	0.118	0.080	0.076	0.101	0.079	0.087	0.112
TFT	no	tuned	0.076	0.083	0.108	0.063	0.056	0.072	0.067	0.081	0.102
TFT	AGE	default	0.102	0.116	0.147	0.106	0.099	0.132	0.115	0.125	0.165
TFT	AGE	tuned	0.071	0.079	0.105	0.087	0.077	0.096	0.075	0.082	0.106
TFT	LC	default	0.110	0.124	0.157	0.130	0.118	0.148	0.140	0.151	0.200
TFT	LC	tuned	0.090	0.100	0.142	0.077	0.072	0.092	0.069	0.077	0.105
TFT	AGE+LC	default	0.147	0.167	0.208	0.124	0.114	0.141	0.133	0.145	0.192
TFT	AGE+LC	tuned	0.088	0.096	0.121	0.094	0.083	0.113	0.078	0.078	0.107
DeepAR	no	default	0.086	0.095	0.118	0.071	0.067	0.084	0.064	0.072	0.105
DeepAR	no	tuned	0.054	0.058	0.077	0.055	0.048	0.061	0.048	0.053	0.073
DeepAR	AGE	default	0.057	0.063	0.080	0.050	0.046	0.058	0.051	0.055	0.079
DeepAR	AGE	tuned	**0.045**	**0.050**	**0.063**	**0.045**	**0.041**	**0.052**	**0.039**	**0.043**	**0.056**
DeepAR	LC	default	0.064	0.069	0.088	0.057	0.049	0.063	0.059	0.065	0.094
DeepAR	LC	tuned	**0.046**	**0.052**	**0.065**	**0.043**	**0.040**	**0.052**	**0.039**	**0.043**	**0.061**
DeepAR	AGE+LC	default	0.059	0.064	0.085	0.054	0.047	0.059	0.063	0.067	0.099
DeepAR	AGE+LC	tuned	**0.048**	0.056	**0.067**	**0.044**	**0.043**	**0.054**	**0.045**	**0.048**	**0.064**

7-Days Horizon

Algorithm	Covariates	Parameters	2014 MAPE	2014 MAE	2014 RMSE	2015 MAPE	2015 MAE	2015 RMSE	2016 MAPE	2016 MAE	2016 RMSE
ARIMA	no	default	**0.135**	**0.197**	**0.250**	0.138	0.179	0.222	0.169	0.172	0.220
ARIMA	no	tuned	**0.135**	**0.197**	**0.250**	0.138	0.179	0.222	0.169	0.172	0.220
SARIMA	no	default	**0.135**	**0.197**	**0.250**	0.138	0.179	0.222	0.169	0.172	0.220
SARIMA	no	tuned	0.154	0.223	0.281	0.138	0.179	0.222	0.169	0.172	0.220
ETS	no	default	**0.135**	**0.197**	**0.250**	0.138	0.179	0.222	0.169	0.172	0.220
ETS	no	tuned	0.154	0.223	0.281	0.138	0.179	0.222	0.169	0.172	0.220
Theta	no	default	**0.135**	**0.197**	**0.250**	0.138	0.179	0.222	0.169	0.172	0.220
Theta	no	tuned	0.154	0.223	0.281	0.138	0.179	0.222	0.169	0.172	0.220
CrostonSBA	no	default	**0.135**	**0.197**	**0.250**	0.138	0.179	0.222	0.169	0.172	0.220
CrostonSBA	no	tuned	**0.135**	**0.197**	**0.250**	0.138	0.179	0.222	0.169	0.172	0.220
NPTS	no	default	0.589	0.928	0.996	0.508	0.722	0.777	0.508	0.534	0.573
NPTS	no	tuned	0.276	0.429	0.581	0.237	0.292	0.374	0.445	0.415	0.471
PatchTST	no	default	0.186	0.265	0.322	0.165	0.208	0.265	0.190	0.180	0.218
PatchTST	no	tuned	0.205	0.269	0.347	0.185	0.207	0.239	0.189	0.171	0.202
Chronos	no	default	0.147	0.216	0.279	0.148	0.198	0.245	0.191	0.193	0.240
Chronos	no	tuned	**0.147**	**0.220**	**0.279**	0.173	0.210	0.269	0.187	0.188	0.234
TFT	no	default	0.173	0.237	0.297	**0.123**	0.158	**0.200**	0.192	0.184	0.234
TFT	no	tuned	0.166	0.221	0.311	0.151	0.193	0.247	0.184	0.167	0.245
TFT	AGE	default	0.187	0.259	0.315	0.158	0.198	0.237	0.176	0.172	0.228
TFT	AGE	tuned	0.177	0.241	0.326	0.163	0.234	0.311	0.179	0.174	0.206
TFT	LC	default	0.238	0.313	0.413	0.228	0.306	0.382	0.166	**0.160**	0.212
TFT	LC	tuned	0.207	0.265	0.398	0.123	0.165	0.206	0.178	0.177	0.224
TFT	AGE+LC	default	0.245	0.330	0.409	0.224	0.305	0.407	0.172	0.165	0.216
TFT	AGE+LC	tuned	0.195	0.254	0.330	**0.112**	**0.151**	0.206	0.212	0.206	0.257
DeepAR	no	default	0.193	0.282	0.345	0.207	0.252	0.308	0.169	0.169	0.219
DeepAR	no	tuned	0.197	0.270	0.312	0.172	0.228	0.249	0.192	0.162	0.231
DeepAR	AGE	default	0.208	0.289	0.363	0.241	0.295	0.367	0.164	0.162	0.211
DeepAR	AGE	tuned	0.195	0.272	0.325	0.128	0.160	**0.197**	**0.159**	**0.153**	**0.203**
DeepAR	LC	default	0.193	0.276	0.334	0.213	0.270	0.364	0.174	0.165	**0.201**
DeepAR	LC	tuned	0.189	0.250	0.319	0.182	0.220	0.292	0.174	0.167	0.210
DeepAR	AGE+LC	default	0.201	0.284	0.355	0.209	0.266	0.345	0.174	0.167	0.209
DeepAR	AGE+LC	tuned	0.210	0.283	0.362	0.127	**0.160**	0.198	**0.164**	0.158	0.213

7-Days Horizon

Algorithm	Covariates	Parameters	2017 MAPE	2017 MAE	2017 RMSE	2018 MAPE	2018 MAE	2018 RMSE	2019 MAPE	2019 MAE	2019 RMSE
ARIMA	no	default	0.152	0.176	0.215	0.167	0.162	0.202	0.137	0.159	0.205
ARIMA	no	tuned	0.152	0.176	0.215	0.167	0.162	0.202	0.137	0.159	0.205
SARIMA	no	default	0.152	0.176	0.215	0.167	0.162	0.202	0.137	0.159	0.205
SARIMA	no	tuned	0.152	0.176	0.215	0.167	0.162	0.202	0.137	0.159	0.205
ETS	no	default	0.152	0.176	0.215	0.167	0.162	0.202	0.137	0.159	0.205
ETS	no	tuned	0.152	0.176	0.215	0.167	0.162	0.202	0.137	0.159	0.205
Theta	no	default	0.152	0.176	0.215	0.167	0.162	0.202	0.137	0.159	0.205
Theta	no	tuned	0.152	0.176	0.215	0.167	0.162	0.202	0.137	0.159	0.205
CrostonSBA	no	default	0.152	0.176	0.215	0.167	0.162	0.202	0.137	0.159	0.205
CrostonSBA	no	tuned	0.152	0.176	0.215	0.167	0.162	0.202	0.137	0.159	0.205
NPTS	no	default	0.560	0.682	0.718	0.499	0.519	0.560	0.578	0.661	0.688
NPTS	no	tuned	0.232	0.268	0.327	0.285	0.256	0.321	0.212	0.237	0.295
PatchTST	no	default	0.225	0.239	0.295	0.160	0.149	0.184	0.239	0.260	0.337
PatchTST	no	tuned	0.198	0.190	0.223	0.159	0.138	0.170	0.156	0.155	0.195
Chronos	no	default	0.161	0.191	0.237	0.183	0.180	0.220	0.146	0.173	0.235
Chronos	no	tuned	0.144	0.160	0.200	0.185	0.178	0.212	0.148	0.169	0.230
TFT	no	default	0.139	0.155	0.188	0.130	0.127	0.162	0.147	0.162	0.196
TFT	no	tuned	**0.131**	**0.149**	**0.183**	0.140	0.139	0.172	0.157	0.188	0.220
TFT	AGE	default	0.141	**0.157**	0.197	**0.119**	0.118	**0.152**	0.150	0.163	0.199
TFT	AGE	tuned	**0.134**	0.166	0.196	0.149	0.135	0.166	0.152	0.169	0.205
TFT	LC	default	0.147	0.165	0.205	0.147	0.144	0.178	0.172	0.187	0.237
TFT	LC	tuned	0.164	0.191	0.230	0.133	0.132	0.168	**0.128**	0.149	**0.186**
TFT	AGE+LC	default	0.168	0.194	0.242	0.141	0.137	0.168	0.175	0.190	0.242
TFT	AGE+LC	tuned	0.174	0.191	0.245	0.136	0.136	0.176	0.157	0.168	0.202
DeepAR	no	default	0.175	0.201	0.238	0.151	0.145	0.179	0.134	0.156	0.212
DeepAR	no	tuned	0.189	0.194	0.250	0.119	**0.112**	**0.142**	0.143	0.165	0.208
DeepAR	AGE	default	0.196	0.225	0.263	0.177	0.174	0.212	0.147	0.173	0.247
DeepAR	AGE	tuned	0.132	0.158	**0.195**	**0.123**	**0.123**	0.150	0.131	**0.152**	**0.195**
DeepAR	LC	default	0.187	0.213	0.251	0.178	0.170	0.207	0.143	0.170	0.248
DeepAR	LC	tuned	0.154	0.174	0.235	0.129	0.125	0.160	0.125	0.152	0.223
DeepAR	AGE+LC	default	0.194	0.221	0.265	0.187	0.177	0.209	0.160	0.186	0.260
DeepAR	AGE+LC	tuned	0.145	0.175	0.210	0.135	0.134	0.163	**0.113**	**0.142**	0.193

**14-Days Horizon**				**2014**			**2015**			**2016**	
Algorithm	Covariates	Parameters	MAPE	MAE	RMSE	MAPE	MAE	RMSE	MAPE	MAE	RMSE
ARIMA	no	default	0.174	**0.268**	0.331	0.189	0.244	0.291	0.209	0.210	0.246
ARIMA	no	tuned	0.174	**0.268**	0.331	0.189	0.244	0.291	0.209	0.210	0.246
SARIMA	no	default	0.174	**0.268**	0.331	0.189	0.244	0.291	0.209	0.210	0.246
SARIMA	no	tuned	**0.187**	0.307	0.419	0.223	0.244	0.291	0.209	0.210	0.246
ETS	no	default	0.174	**0.268**	0.331	0.189	0.244	0.291	0.209	0.210	0.246
ETS	no	tuned	**0.187**	0.307	0.419	0.223	0.244	0.291	0.209	0.210	0.246
Theta	no	default	0.174	**0.268**	0.331	0.189	0.244	0.291	0.209	0.210	0.246
Theta	no	tuned	**0.187**	0.307	0.419	0.223	0.244	0.291	0.209	0.210	0.246
CrostonSBA	no	default	0.174	**0.268**	0.331	0.189	0.244	0.291	0.209	0.210	0.246
CrostonSBA	no	tuned	0.174	**0.268**	0.331	0.189	0.244	0.291	0.209	0.210	0.246
NPTS	no	default	0.634	1.007	1.063	0.515	0.741	0.792	0.542	0.577	0.608
NPTS	no	tuned	0.316	0.464	0.620	0.238	0.296	0.381	0.422	0.412	0.472
PatchTST	no	default	0.230	0.347	0.425	0.177	0.235	0.309	0.228	0.228	0.278
PatchTST	no	tuned	0.243	0.363	0.428	**0.154**	**0.211**	**0.264**	0.206	0.211	0.254
Chronos	no	default	0.192	0.306	0.391	0.190	0.256	0.310	0.232	0.236	0.277
Chronos	no	tuned	0.193	0.302	0.376	0.227	0.293	0.346	0.233	0.240	0.256
TFT	no	default	0.227	0.316	0.399	**0.135**	**0.177**	**0.221**	0.168	**0.171**	0.238
TFT	no	tuned	0.220	0.328	0.381	0.185	0.223	0.295	0.168	**0.171**	0.238
TFT	AGE	default	0.230	0.326	0.392	0.153	0.195	0.244	0.173	0.181	0.240
TFT	AGE	tuned	0.251	0.392	0.453	0.208	0.240	0.295	0.173	0.192	0.248
TFT	LC	default	0.253	0.347	0.476	0.268	0.353	0.432	**0.155**	**0.157**	**0.214**
TFT	LC	tuned	0.304	0.406	0.519	0.197	0.263	0.298	0.226	0.219	0.254
TFT	AGE+LC	default	0.313	0.435	0.535	0.258	0.351	0.448	0.172	0.170	0.227
TFT	AGE+LC	tuned	0.256	0.342	0.450	0.185	0.249	0.315	0.239	0.244	0.298
DeepAR	no	default	0.201	0.326	0.467	0.228	0.293	0.390	0.215	0.217	0.263
DeepAR	no	tuned	**0.173**	0.272	**0.358**	0.170	0.231	0.308	0.221	0.216	0.281
DeepAR	AGE	default	0.203	**0.285**	0.360	0.257	0.335	0.454	0.189	0.189	0.235
DeepAR	AGE	tuned	0.199	0.310	0.382	0.193	0.260	0.329	**0.169**	0.177	0.216
DeepAR	LC	default	0.218	0.323	0.410	0.249	0.332	0.436	0.208	0.200	0.258
DeepAR	LC	tuned	0.210	0.296	**0.307**	0.258	0.305	0.373	0.193	0.206	**0.226**
DeepAR	AGE+LC	default	0.244	0.352	0.428	0.253	0.349	0.460	0.226	0.225	0.271
DeepAR	AGE+LC	tuned	0.227	0.308	0.363	0.211	0.275	0.324	0.195	0.191	0.263

**14-Days Horizon**				**2017**			**2018**			**2019**	
Algorithm	Covariates	Parameters	MAPE	MAE	RMSE	MAPE	MAE	RMSE	MAPE	MAE	RMSE
ARIMA	no	default	0.213	0.245	0.281	0.177	0.173	0.206	0.150	0.169	0.213
ARIMA	no	tuned	0.213	0.245	0.281	0.177	0.173	0.206	0.150	0.169	0.213
SARIMA	no	default	0.213	0.245	0.281	0.177	0.173	0.206	0.150	0.169	0.213
SARIMA	no	tuned	0.213	0.245	0.281	0.177	0.173	0.206	0.150	0.169	0.213
ETS	no	default	0.213	0.245	0.281	0.177	0.173	0.206	0.150	0.169	0.213
ETS	no	tuned	0.213	0.245	0.281	0.177	0.173	0.206	0.150	0.169	0.213
Theta	no	default	0.213	0.245	0.281	0.177	0.173	0.206	0.150	0.169	0.213
Theta	no	tuned	0.213	0.245	0.281	0.177	0.173	0.206	0.150	0.169	0.213
CrostonSBA	no	default	0.213	0.245	0.281	0.177	0.173	0.206	0.150	0.169	0.213
CrostonSBA	no	tuned	0.213	0.245	0.281	0.177	0.173	0.206	0.150	0.169	0.213
NPTS	no	default	0.531	0.658	0.689	0.492	0.522	0.563	0.567	0.649	0.678
NPTS	no	tuned	0.219	0.269	0.332	0.291	0.264	0.328	0.201	0.225	0.282
PatchTST	no	default	0.229	0.252	0.315	0.189	0.182	0.219	0.303	0.320	0.394
PatchTST	no	tuned	0.193	0.229	0.260	0.163	0.159	0.193	0.215	0.236	0.276
Chronos	no	default	0.225	0.270	0.322	0.220	0.224	0.274	0.198	0.233	0.302
Chronos	no	tuned	0.164	0.191	0.228	0.195	0.202	0.243	0.181	0.203	0.275
TFT	no	default	0.145	**0.163**	**0.222**	0.154	0.153	**0.180**	0.189	0.197	0.247
TFT	no	tuned	0.145	**0.163**	**0.222**	0.154	0.153	**0.180**	0.189	0.197	0.247
TFT	AGE	default	0.175	0.202	0.265	**0.149**	0.148	0.180	0.173	0.184	0.239
TFT	AGE	tuned	**0.148**	**0.150**	**0.199**	0.155	**0.142**	0.182	0.172	0.187	0.223
TFT	LC	default	**0.145**	0.161	0.210	**0.137**	0.143	0.187	0.183	0.197	0.258
TFT	LC	tuned	0.149	0.180	0.229	0.160	**0.145**	0.196	0.159	0.186	**0.187**
TFT	AGE+LC	default	0.171	0.201	0.263	0.151	0.151	0.191	0.184	0.198	0.264
TFT	AGE+LC	tuned	0.182	0.174	0.254	0.142	0.149	0.188	0.170	0.208	0.217
DeepAR	no	default	0.211	0.245	0.297	0.162	0.163	0.199	0.142	**0.164**	0.214
DeepAR	no	tuned	0.193	0.232	0.271	0.179	0.180	**0.164**	**0.146**	0.169	**0.209**
DeepAR	AGE	default	0.224	0.264	0.321	0.195	0.197	0.237	0.166	0.195	0.258
DeepAR	AGE	tuned	0.159	0.192	0.238	0.157	0.160	0.184	0.147	**0.167**	0.205
DeepAR	LC	default	0.233	0.279	0.341	0.201	0.200	0.251	0.182	0.212	0.283
DeepAR	LC	tuned	0.169	0.205	0.313	0.152	0.155	0.187	0.147	0.164	0.236
DeepAR	AGE+LC	default	0.241	0.293	0.361	0.230	0.226	0.270	0.182	0.213	0.288
DeepAR	AGE+LC	tuned	0.173	0.203	0.238	0.167	0.157	0.188	**0.129**	0.203	0.260

21-Days Horizon			2014			2015			2016		
Algorithm	Covariates	Parameters	MAPE	MAE	RMSE	MAPE	MAE	RMSE	MAPE	MAE	RMSE
ARIMA	no	default	0.205	0.330	0.399	0.230	0.297	0.359	0.244	0.254	0.296
ARIMA	no	tuned	0.205	0.330	0.399	0.230	0.297	0.359	0.244	0.254	0.296
SARIMA	no	default	0.205	0.330	0.399	0.230	0.297	0.359	0.244	0.254	0.296
SARIMA	no	tuned	0.226	0.390	0.508	0.245	0.307	0.413	0.277	0.257	0.296
ETS	no	default	0.205	0.330	0.399	0.230	0.297	0.359	0.244	0.254	0.296
ETS	no	tuned	0.226	0.390	0.508	0.245	0.307	0.413	0.277	0.257	0.296
Theta	no	default	0.205	0.330	0.399	0.230	0.297	0.359	0.244	0.254	0.296
Theta	no	tuned	0.226	0.390	0.508	0.245	0.307	0.413	0.277	0.257	0.296
CrostonSBA	no	default	0.205	0.330	0.399	0.230	0.297	0.359	0.244	0.254	0.296
CrostonSBA	no	tuned	0.205	0.330	0.399	0.230	0.297	0.359	0.244	0.254	0.296
NPTS	no	default	0.625	1.019	1.067	0.583	0.845	0.892	0.537	0.577	0.603
NPTS	no	tuned	0.405	0.618	0.776	0.249	0.311	0.392	0.418	0.417	0.477
PatchTST	no	default	0.285	0.449	0.531	0.196	0.264	0.340	0.266	0.276	0.328
PatchTST	no	tuned	0.272	0.463	0.539	0.174	0.255	0.293	0.211	0.227	0.293
Chronos	no	default	0.240	0.400	0.478	0.228	0.314	0.385	0.275	0.292	0.348
Chronos	no	tuned	0.237	0.389	0.475	0.237	0.303	0.362	0.236	0.246	0.288
TFT	no	default	0.219	0.332	0.407	0.145	0.189	0.232	0.203	0.212	0.291
TFT	no	tuned	0.247	0.374	0.444	0.150	0.188	0.235	0.203	0.212	0.291
TFT	AGE	default	0.214	0.323	0.394	0.155	0.193	0.241	0.215	0.231	0.296
TFT	AGE	tuned	0.246	0.386	0.453	0.194	0.244	0.312	0.215	0.231	0.296
TFT	LC	default	0.280	0.416	0.568	0.293	0.387	0.480	0.165	0.176	0.222
TFT	LC	tuned	0.338	0.492	0.591	0.233	0.330	0.404	0.232	0.242	0.299
TFT	AGE+LC	default	0.306	0.471	0.615	0.299	0.411	0.519	0.187	0.191	0.247
TFT	AGE+LC	tuned	0.278	0.416	0.474	0.247	0.314	0.404	0.239	0.240	0.323
DeepAR	no	default	0.248	0.407	0.552	0.260	0.342	0.424	0.218	0.226	0.276
DeepAR	no	tuned	0.217	0.366	0.472	0.298	0.379	0.503	0.237	0.250	0.291
DeepAR	AGE	default	0.266	0.399	0.483	0.287	0.382	0.496	0.222	0.228	0.272
DeepAR	AGE	tuned	0.198	0.282	0.394	0.208	0.282	0.355	0.189	0.192	0.231
DeepAR	LC	default	0.270	0.419	0.531	0.295	0.404	0.523	0.244	0.249	0.312
DeepAR	LC	tuned	0.290	0.437	0.527	0.248	0.335	0.435	0.197	0.201	0.309
DeepAR	AGE+LC	default	0.297	0.448	0.555	0.274	0.383	0.507	0.257	0.267	0.328
DeepAR	AGE+LC	tuned	0.258	0.386	0.487	0.245	0.318	0.389	0.221	0.232	0.290

21-Days Horizon			2017			2018			2019		
Algorithm	Covariates	Parameters	MAPE	MAE	RMSE	MAPE	MAE	RMSE	MAPE	MAE	RMSE
ARIMA	no	default	0.184	0.219	0.276	0.209	0.208	0.240	0.180	0.200	0.240
ARIMA	no	tuned	0.184	0.219	0.276	0.209	0.208	0.240	0.180	0.200	0.240
SARIMA	no	default	0.184	0.219	0.276	0.209	0.208	0.240	0.180	0.200	0.240
SARIMA	no	tuned	0.184	0.219	0.276	0.209	0.208	0.240	0.180	0.200	0.240
ETS	no	default	0.184	0.219	0.276	0.209	0.208	0.240	0.180	0.200	0.240
ETS	no	tuned	0.184	0.219	0.276	0.209	0.208	0.240	0.180	0.200	0.240
Theta	no	default	0.184	0.219	0.276	0.209	0.208	0.240	0.180	0.200	0.240
Theta	no	tuned	0.184	0.219	0.276	0.209	0.208	0.240	0.180	0.200	0.240
CrostonSBA	no	default	0.184	0.219	0.276	0.209	0.208	0.240	0.180	0.200	0.240
CrostonSBA	no	tuned	0.184	0.219	0.276	0.209	0.208	0.240	0.180	0.200	0.240
NPTS	no	default	0.562	0.709	0.748	0.576	0.617	0.655	0.554	0.640	0.677
NPTS	no	tuned	0.243	0.308	0.353	0.290	0.267	0.334	0.193	0.217	0.277
PatchTST	no	default	0.212	0.244	0.305	0.206	0.202	0.236	0.343	0.352	0.429
PatchTST	no	tuned	0.192	0.222	0.262	0.182	0.188	0.231	0.260	0.272	0.316
Chronos	no	default	0.203	0.253	0.314	0.295	0.313	0.372	0.290	0.339	0.423
Chronos	no	tuned	0.160	0.195	0.254	0.238	0.237	0.271	0.194	0.216	0.268
TFT	no	default	0.147	0.169	0.222	0.174	0.179	0.220	0.186	0.197	0.245
TFT	no	tuned	0.147	0.169	0.222	0.174	0.179	0.220	0.186	0.197	0.245
TFT	AGE	default	0.178	0.201	0.291	0.175	0.179	0.215	0.193	0.204	0.257
TFT	AGE	tuned	0.152	0.191	0.263	0.168	0.165	0.206	0.196	0.208	0.273
TFT	LC	default	0.133	0.154	0.197	0.148	0.158	0.204	0.190	0.203	0.283
TFT	LC	tuned	0.153	0.181	0.311	0.164	0.167	0.192	0.186	0.207	0.283
TFT	AGE+LC	default	0.176	0.217	0.287	0.171	0.175	0.213	0.204	0.215	0.301
TFT	AGE+LC	tuned	0.168	0.192	0.243	0.177	0.182	0.214	0.211	0.211	0.279
DeepAR	no	default	0.217	0.264	0.340	0.178	0.185	0.227	0.166	0.192	0.250
DeepAR	no	tuned	0.163	0.175	0.233	0.168	0.171	0.210	0.153	0.172	0.216
DeepAR	AGE	default	0.250	0.311	0.393	0.240	0.249	0.288	0.177	0.210	0.283
DeepAR	AGE	tuned	0.182	0.227	0.283	0.137	0.151	0.192	0.161	0.184	0.219
DeepAR	LC	default	0.258	0.323	0.399	0.251	0.255	0.303	0.210	0.248	0.327
DeepAR	LC	tuned	0.198	0.254	0.375	0.173	0.185	0.221	0.167	0.188	0.280
DeepAR	AGE+LC	default	0.273	0.345	0.429	0.266	0.268	0.316	0.207	0.245	0.326
DeepAR	AGE+LC	tuned	0.176	0.228	0.287	0.157	0.167	0.200	0.139	0.165	0.212

Table S2 – Performance of the algorithms within the benchmark for four different days on the forecasting horizon.

The performance of the algorithms is expressed by the Mean-Absolute-Percentage-Error (MAPE), Mean-Absolute-Error (MAE), and Root-Mean-Squared-Error (RMSE) for the different validation years. The best performance per column is highlighted in blue, orange highlights the second, and purple is the third-best error score achieved.

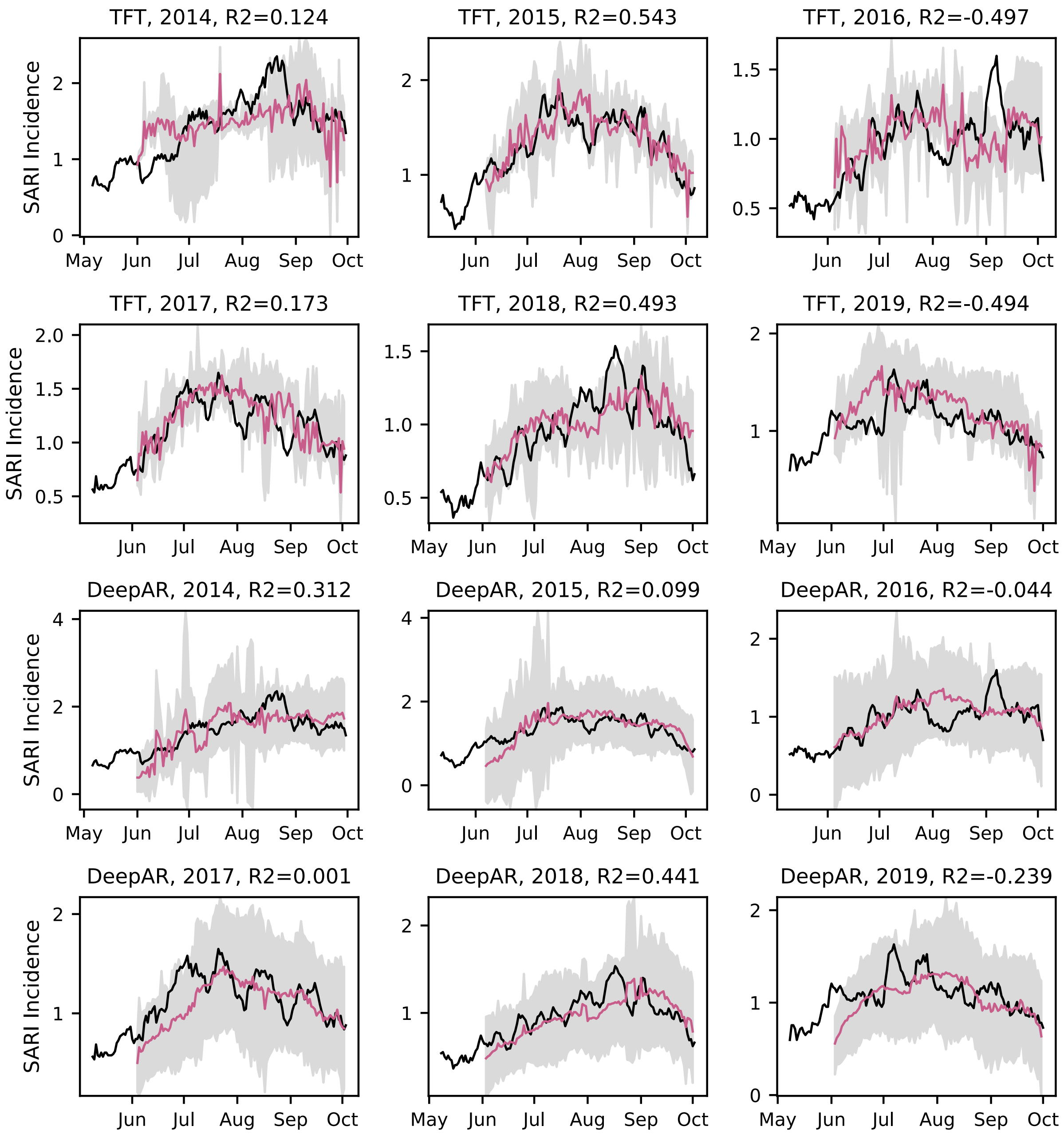

Figure S5 – Point and probabilistic forecast of the Week 2 forecast compared to the true SARI incidence.

Forecasts were extracted from the 1-day walk-forward validation, and the temporal resolution for the point and probabilistic forecasts were changed from daily to weekly. The pink line represents the point forecast, and the shaded area is the 90% confidence interval derived from the probabilistic forecasting as estimated during the model training. The black line shows the truly observed SARI incidence. The validation year is shown in the upper left corner of the corresponding subfigure.

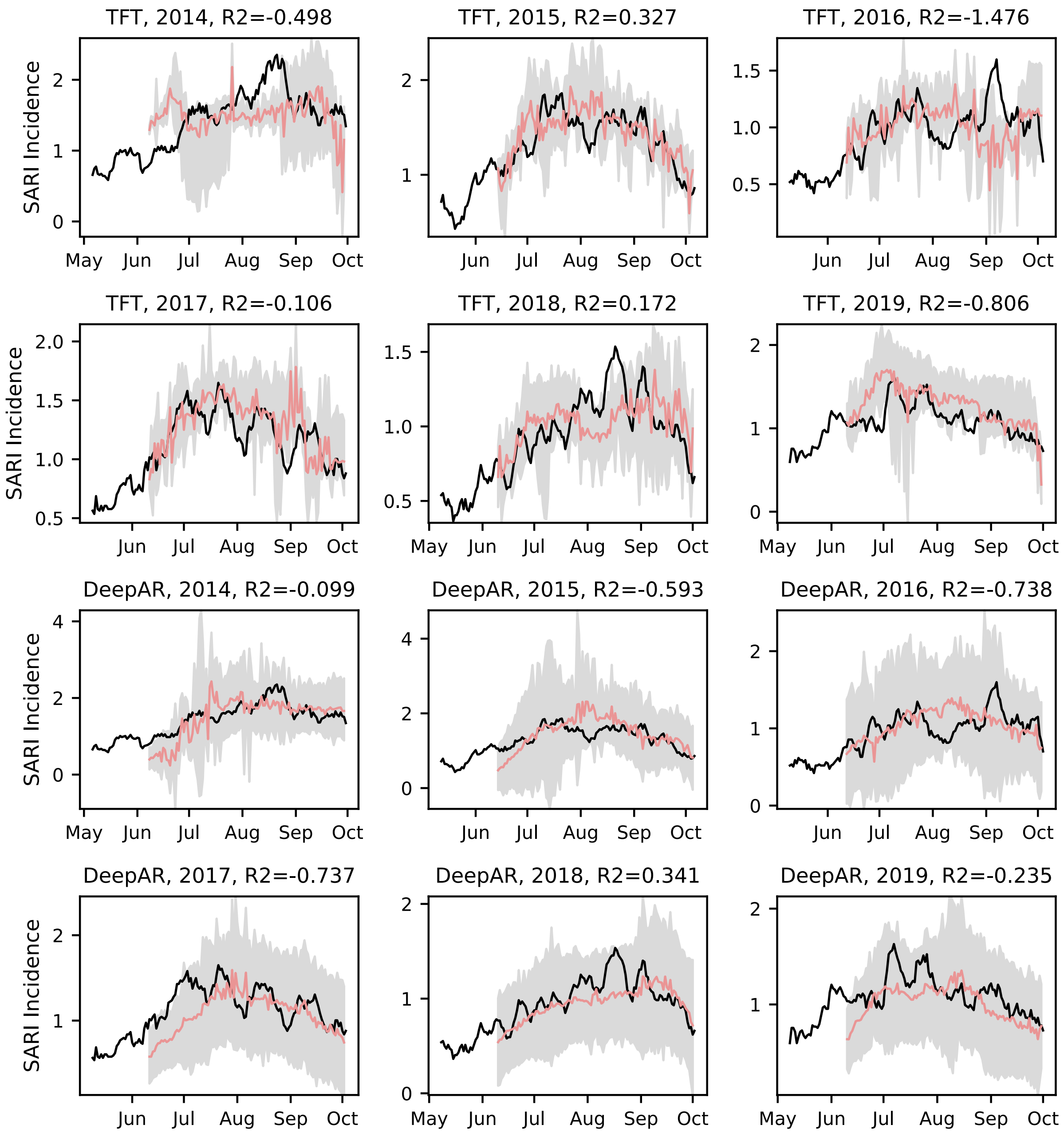

Figure S6 – Point and probabilistic forecast of the Week 3 forecast compared to the true SARI incidence.

Forecasts were extracted from the 1-day walk-forward validation, and the temporal resolution for the point and probabilistic forecasts were changed from daily to weekly. The salmon-colored line represents the point forecast, and the shaded area is the 90% confidence interval derived from the probabilistic forecasting estimated during the model training. The black line shows the truly observed SARI incidence. The validation year is shown in the upper left corner of the corresponding subfigure.
